# Exploring Adaptive Cycling Interventions for Young People with Disability: An Online Survey of Providers in Australia

**DOI:** 10.3390/jcm12175523

**Published:** 2023-08-25

**Authors:** John J. Carey, Rachel Toovey, Alicia J. Spittle, Christine Imms, Nora Shields

**Affiliations:** 1 Department of Physiotherapy, University of Melbourne, Carlton, Melbourne, VIC 3053, Australia; r.toovey@unimelb.edu.au (R.T.);; 2 Murdoch Children’s Research Institute, Parkville, Melbourne, VIC 3052, Australia; 3 Department of Paediatrics, University of Melbourne, Parkville, Melbourne, VIC 3052, Australia; 4 Olga Tennison Autism Research Centre, School of Psychology and Public Health, La Trobe University, Bundoora, Melbourne, VIC 3086, Australia

**Keywords:** adaptive cycling, disability, online survey, current practices, participation

## Abstract

Adapted cycles offer young people with disability a fun way to participate in over-ground cycling, but little is known about current practices to train and sustain cycling in this group. This study aimed to describe interventions used to introduce adaptive cycling to young people with disability and explore barriers and facilitators to adapted cycle use. A cross-sectional online survey was distributed among Australian allied health, education and recreation providers through targeted advertizing and snowball methods. Data were analysed using mixed methods and reporting was guided by the CHERRIES and CROSS checklists. There were 107 respondents with n = 90 (84.1%) who fully completed the survey. Respondents worked with riders who had cerebral palsy, neurodevelopmental disabilities and movement impairments. Adaptive cycling interventions were customized according to a rider’s goals, needs and resourcing. The training of cycling skills included “an eclectic mix” of experiential learning, individual goals, task-specific training and holistic practice models. Diverse factors impacted cycling participation, with opportunities reliant on access to a supportive environment, including a suitable adapted cycle. This study found that providers viewed adaptive cycling as a therapeutic or active leisure experience within protected traffic-free environments. Strategies to extend adaptive cycling opportunities into the community are required.

## 1. Introduction

Bicycle riding is one of Australia’s most popular physical activities with approximately 70% of 2 to 17 year olds cycling annually [1]. It is a popular activity goal for young people with neurodevelopmental disability, although fewer ambulant young people with cerebral palsy (3–51%) [2], autism spectrum disorder (20–33%) [3,4] and Down syndrome (9–36%) [5,6] learn how to cycle a two-wheel bicycle. The motor demands required to ride a standard two-wheel bicycle mean prospective riders with impaired balance or trunk control, and those who are non-ambulant or require mobility aids or guided assistance, often need adapted cycles to safely take part in cycling. This has led many young people with disability and their families to explore adapted cycling options through learn-to-ride programs [7,8,9] and try-a-bike initiatives [10,11]. To date, research has focused on cycling for physical activity, fitness, and training 2-wheel bicycle skills [12,13]. Less is known about approaches to training cycle skills for riders who are new to adaptive cycling and what factors are important to sustaining cycling participation in the home, school or community environments.

Adaptive cycling interventions are beneficial for young people with disability, including those who walk with support, with studies showing improvements in gross motor function, lower limb muscle strength, physical activity and the attainment of cycling goals [12]. Adapted cycles include modified or customized cycling equipment with specialized features, which cater for individual needs. The Union Cycliste Internationale, which governs Paralympic categories of cycling, recognizes adapted cycles to include tricycles, handcycles, tandem and customized two-wheel bicycles [14]; however, many other adapted cycles are available, including hybrids (e.g., semi-recumbent bikes), e-cycles (e.g., power-assisted) and companion cycles. Bike frames can be upright, recumbent or semi-recumbent, which is important for a rider’s centre of mass, stability and proposed bike use [14]. Young people with disability face additional challenges to starting and sustaining cycling participation such as finding a suitable bike [15,16] and having access to inclusive cycling opportunities [11]. Understanding facilitators and barriers to cycling is useful when a rider’s goal relates to participation [17]. To date, studies on the barriers and facilitators to cycling participation have largely focused on people without disability and two-wheel bicycle use for active transport [18]. There has been limited research on barriers and facilitators for riders with disability and people who have goals to ride for active leisure.

Organized cycling opportunities are often facilitated by different members of a young person’s formal support network [10,13]. Cycling ‘providers’ include allied health, education, sport and recreation personnel who have the common objective of supporting a rider and family’s cycling goal. Cycling goals are often set in early childhood [2,19]; however, some young people with disabilities only discover cycling during adulthood [11]. Allied health therapists often support the assessment of function, posture and posture support needs relative to an adapted cycle [10], while therapists, teachers, cycling instructors and support workers help to support early skill development and the use of adapted cycles [20,21]. Descriptions of adaptive cycling interventions used by Australian providers are lacking, and understanding the ‘current practice’ forms an important step to developing new interventions [22]. The research question posed by this study was “how do therapists, teachers and other cycling providers describe their current practices for implementing adaptive cycling interventions for young people with disability aged 2 to 30 years?”.

The study aims were:

- To describe adaptive cycling interventions delivered by allied health therapists, teachers and sports and recreation providers using the template for intervention description and replication (TIDieR) checklist [23].
- To explore providers’ perspectives for recommending an adaptive cycling intervention, and the barriers and facilitators for using an adapted cycle.

## 2. Materials and Methods

### 2.1. Study Design

A study-specific, cross-sectional online survey was completed. The survey (Supplementary File S1) was hosted openly on the web-based application Research Electronic Data Capture (REDCap) [24]. This method allowed for data collection about current practices from diverse sectors (e.g., recreation, education, allied health) nationwide. Close and open-ended questions enabled respondents to share descriptive and reflective perspectives. The Checklist for Reporting of Internet E-Surveys (CHERRIES) [25] and Consensus-Based Checklist for Reporting of Survey Studies (CROSS) [26] guided the reporting of the study’s design and findings (Supplementary File S2).

The study received ethics approval from the University of Melbourne’s Human Research Ethics Committee (reference number: 20449). Informed consent was obtained electronically after eligibility screening and before survey commencement. Respondents could choose to remain fully anonymous or leave their email address to receive a copy of the study results.

### 2.2. Survey Development, Content and Pre-Testing

The survey was adapted from a previous survey on bicycle training in cerebral palsy [13] and further expanded in consultation [27] with industry stakeholders (Supplementary File S3) [28]. It contained 82 questions (69 close-ended, 7 descriptive open-ended (i.e., list or name) and 6 reflective open-ended (i.e., text-based reflection)) divided over five sections: (1) provider demographics; (2) networks and roles; (3) early rider involvement; (4) riders’ needs, goals and abilities and (5) cycling opportunities. Participants were encouraged to share their ‘typical practice’, including pre-pandemic approaches to cycling. Branching logic tailored a respondent’s survey path based on prior responses. Questions on training parameters for adaptive cycling skills were mapped to the 12-item TIDieR checklist [23]. This checklist describes why, what, where, who and how interventions are delivered. Perspectives were sought with regard to the age to start riding, rationale, goals and barriers as well as facilitators to adaptive cycling.

Five professionals with backgrounds in physiotherapy (n = 3), education (n = 1), occupational therapy (n = 1) and one layperson (n = 1) pre-tested the survey for usability and offered feedback on time to complete, wording and accessibility. Participants took 10–20 min (average: 15.3 min) to complete the pilot survey. Changes to the survey’s layout and wording were incorporated following pre-testing.

### 2.3. Participant Eligibility and Recruitment

The study sought allied health professionals (physiotherapists, occupational therapists, exercise physiologists), educators (teachers and teacher aides), cycling coaches and personnel/volunteers working in the sport and recreation sector. We have referred to this collective cohort as ‘providers’. Providers were eligible if they had previous experience supporting children or young people (2–30 years old) to use an adapted cycle. Other eligibility criteria included:

- A recognized qualification and/or registration within their respective area of practice;
- Experience leading adaptive cycling interventions or training in Australia;
- Aged > 18 years; and
- Able to complete a survey in English.

Convenience sampling was used to recruit participants through targeted advertisement and snowballing. This included sharing social media posts and email correspondence (Supplementary File S4) with 14 target organizations, identified through an interest search, who represented: (i) national professional bodies; (ii) affiliated associations; (iii) sport and recreation stakeholders; (iv) research networks and (v) service providers. Key contacts from each respective organization were invited to share project information at two time-points. Snowball sampling meant that interested parties could share the recruitment material with potential participants in their network. Based upon our previous work [13], we anticipated recruiting approximately 150 to 200 participants across Australia. Respondents were not re-imbursed for their time.

### 2.4. Management of Responses

**Figure 1:**
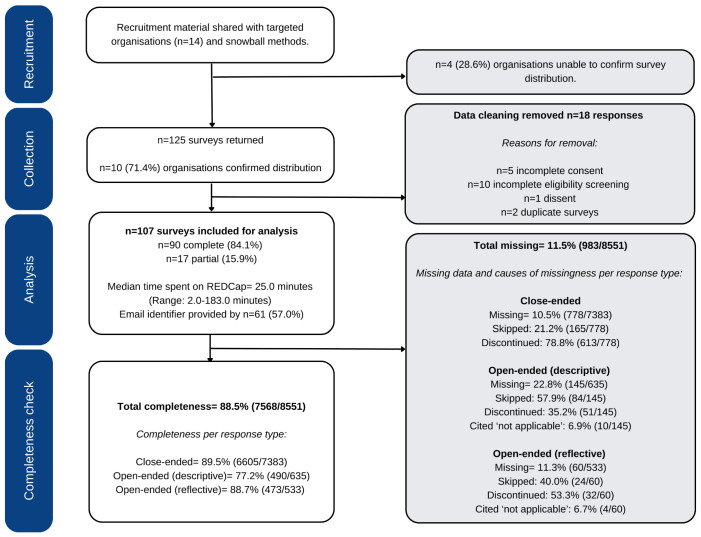
Recruitment and study flowchart.

Data cleaning removed surveys with incomplete screening/consent, ineligible and duplicate responses (see Figure 1). Cases of multiple participation were partially prevented by cross-checking timestamps, demographics (e.g., job title, practice location and experience) and, where available, email identifiers.

### 2.5. Data Analysis

Multiple analysis methods were used to cater for both quantitative and qualitative data. De-identified datasheets were exported to either IBM Statistical Package for Social Sciences (SPSS) Version 28 for close-ended responses or Microsoft Excel for open-ended responses. Given the study’s exploratory focus, we reported responses from all respondents, including partial responders.

Survey completion rate was calculated using the ratio of completed surveys (i.e., respondents who finished Section 5) divided by screened participants who provided informed consent [25]. The completeness rate was calculated after all surveys were submitted by counting the proportion of mandatory questions answered by respondents. A Pearson chi-squared test was used to compare completion rates among three provider groupings (i.e., physiotherapists, occupational therapists and others).

Quantitative data were analysed using descriptive statistics. Close-ended questions were reported as either the proportion of respondents (single-choice questions) or proportion of responses (multi-response questions). To simplify reporting, categorical responses relating to importance (not important at all, not important, neutral, important, extremely important) were collapsed into three (not important, neutral, important) categories. Readers can refer to Supplementary File S5 for original responses.

Open-ended ‘reflective’ questions were analysed using qualitative content analysis [29]. This analysis also involved the three phases of planning, organizing and reporting (see Supplementary File S6 for audit trail overview).

First, researcher JJC transcribed the de-identified responses onto an Excel spreadsheet. Responses were read line by line several times, and notes were scribed in an adjacent column. Familiarization revealed respondents offered diverse response types, including short narratives, bullet points and perspectives in sentence or word form. An inductive approach [29] was chosen to cater for the relative richness of textual responses. It is a useful method for catering with exploratory research or areas where limited research has been performed, such as adaptive cycling. The sentences of text or key words linked to the research question became the unit of analysis.

Once early immersion was completed, researcher JJC re-read the raw data and generated a 1 to 3 word ‘open code’ for each unit of analysis. Open codes were closely connected to the respondent’s words (i.e., manifest coding) and drew upon the researchers’ analytic lens (i.e., pragmatic interpretivist lens). Reflexivity and co-construction of codes (with a second researcher, NS) aided the authenticity of coding.

Codes were grouped into columns based on similarities and differences (organizing). A coding tree (Supplementary File S6) illustrates the abstraction process and the Table facilitated discussion between the two researchers (JJC and NS) and industry stakeholders. This led to further iterations of codes, groupings and preparation for reporting.

Descriptive open-ended responses were analysed through quantitative content analysis. Similar to Elo and Kynagas’ qualitative content analysis approach [29], one researcher (JJC) developed codes based on the respondents’ own words (planning), grouped the codes based on similarities and differences (organizing) and counted the number of codes within each category to decipher the frequency of responses.

## 3. Results

A total of 107 eligible responses were received between March 2021 and November 2021 (Figure 1). The survey completion rate was 84.1% (n = 90) and an overall completeness check found that 88.5% of mandatory questions were answered. There were no statistical differences in survey completion (Pearson chi-squared value: 0.6) between the three provider groups.

### 3.1. Respondent Demographics

Over ten disciplines were represented (Table 1); however, the majority of respondents were allied health therapists who worked in community settings (i.e., allied health organization or private practice) or special education. Providers acquired training in adaptive cycling through informal education (75.8%), formal training (12.8%) and self-directed learning (11.4%). Informal education included ‘on the job’ learning, peer-to-peer support and engagement with equipment suppliers and local knowledge champions (e.g., specialist service or tertiary sector outreach). Providers commented that there was a scarcity of formal education opportunities and learning was often acquired through “trial and error” with riders, suppliers and peers.

### 3.2. Respondent Roles

**Figure 2:**
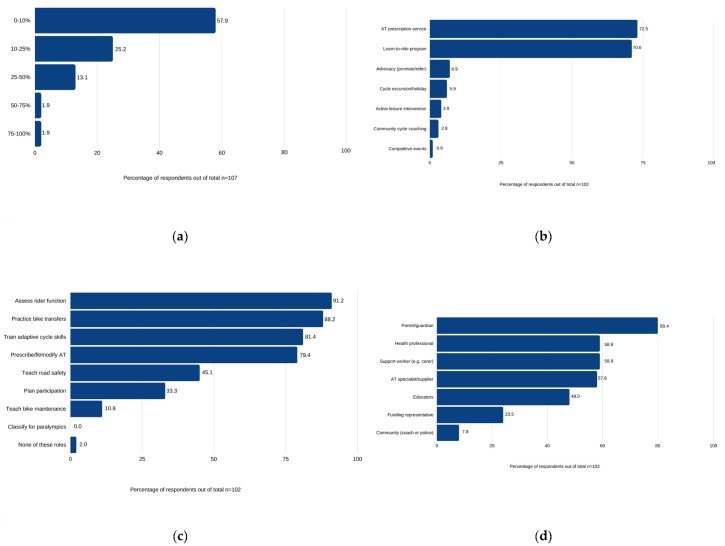
Percentage of respondents involved in adaptive cycling intervention roles/delivery (note: respondents could select multiple response options for (**b**–**d**)): (**a**) estimated workload related to adaptive cycling provision; (**b**) cycling intervention(s) delivered; (**c**) role(s) in cycling intervention delivery; (**d**) support network collaboration.

Adaptive cycling interventions contributed to ≤25% of daily workload for most providers (83.1%) (Figure 2a). Almost all providers played a role (Figure 2c) in rider assessment (91.2%), bike selection (79.4%), transfer practice (88.2%) and skills training (81.4%). Fewer providers were involved in follow-up interventions such as road safety education (45.1%), community participation (33.3%) and bike maintenance (10.8%). Collaboration with the riders’ wider support network (Figure 2d) consisted of immediate (parents, carers and support workers) and formal (educators and health professionals) supports. The mentorship of others was offered by 74.5% of respondents and involved training immediate caregivers and more junior colleagues on operating an adapted cycle and performing safe transfers. Open-ended responses emphasized the role of caregivers in leading community-based practice after the cessation of formalized intervention.

### 3.3. Characteristics of Young Riders with Disability

Providers commonly trained novice riders who lived with cerebral palsy, neurodevelopmental disabilities and neurological conditions (Table 2). Adapted cycles enhanced cycling opportunities and comfort for those with different disabilities. Providers perceived young people would start riding at a mean age of 6.2 years (standard deviation 3.9; range: 3 years–30 years) but this relied on a rider or family’s goals, the awareness that cycling “was an option” and priorities for other mobility or leisure equipment.

### 3.4. Parameters for Beginner Adaptive Cycle Interventions

Respondents primarily catered for beginner riders (89.6%) and focused on skill development (74.2%). As a result, we mapped parameters for training beginner skills in adaptive cycling to the TIDieR checklist [23] (Table 3).

#### 3.4.1. Providers’ Rationale and Goals

**Figure 3:**
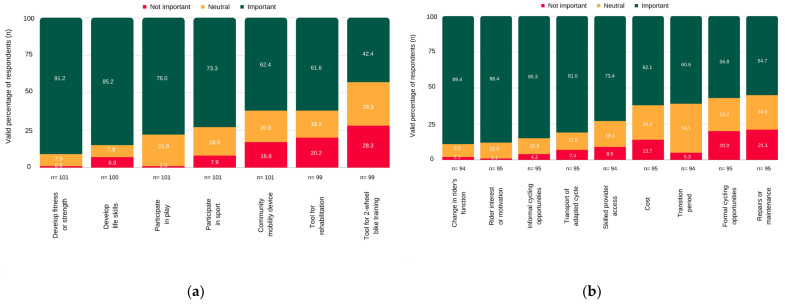
Rating of importance for hypothesized factors to: (**a**) start (providers’ goal) or (**b**) stop adaptive cycling.

Providers recommended adaptive cycling to address therapeutic or learning goals (Figure 3a), to “set the [rider] up for [cycling] success” or in response to a young person’s “self-determined goal”. Cycling was valued as a fun social activity that could improve fitness (e.g., cardiovascular, strength), active leisure participation and develop rider independence.

#### 3.4.2. Essential Elements and Training Approaches

Interventions were customized to a rider’s “unique needs” and usually included goal setting, assessment, the exploration of assistive technologies, early skills development, training immediate supports and practice in meaningful settings. Over 65% of respondents did not have a specific approach to training adaptive cycling skills. Those who usually did, drew upon an “eclectic mix” of frameworks and theories (n = 15 identified). Most commonly, their approach was strength-based, goal-directed and graded to the riders’ ability level to ensure a sense of “success”. Task-specific training (partial and whole) and cognitive-orientated approaches (Cognitive Orientation to daily Occupational Performance (CO-OP) Approach [35]) featured heavily. Providers valued experiential learning opportunities such as trialling different bikes and routine-based practice.

#### 3.4.3. Equipment and Materials

**Figure 4:**
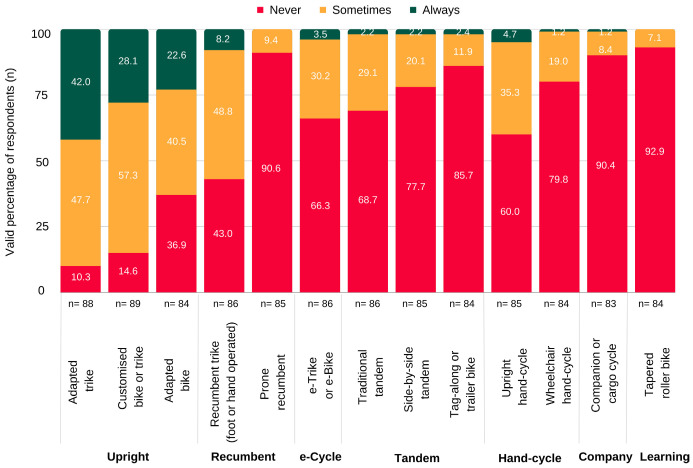
Frequency of use of different adapted cycle types in practice.

Cycling assistive technology was perceived by providers to offer riders “a solution to their particular [physical and safety] needs” and helped “make [cycling] accessible”. Most providers were confident “it is possible to find [a bike] to suit everyone” and “adaptations” existed to support most disabilities. Upright tricycles and modified bicycles were most frequently used in practice (Figure 4), followed by recumbent bikes and handcycles. Commercially available upright tricycles were favoured in school settings where they were “flexible to multiple student’s needs” and offered “easily modifiable [features which could]… be adjusted as skills improve”. Recumbent tricycles, handcycles and e-cycles were more commonly used by providers supporting young adults with spinal cord injury and traumatic brain injury and tandem bikes for people with visual or cognitive impairments.

Other equipment and resources reported to maximize a riders’ participation, safety and function during formal training are listed in Supplementary File S5.

#### 3.4.4. Training Dose

Providers typically trained adaptive cycling skills over several weeks and practiced “foundational” component skills (e.g., transferring on/off the bike, pedalling, steering, braking) in traffic-free environments (Table 4).

**Table 4:** Frequency, intensity, time and type of adaptive cycling (‘FITT’ principles).

FITT Domain	Response	*n*	%
Frequency ^(a)^	Over several weeks	47	52.2
	On request	32	35.6
	Once-off	22	24.2
	3–5 days	12	13.3
	Other	10	11.1
Intensity ^(b)^	Skills focus	66	74.2
	Low intensity	7	7.9
	Moderate intensity	13	14.6
	Moderate to vigorous	3	3.4
Time/duration ^(b)^	<20 min	6	6.7
	30 min	29	32.6
	45 min	28	31.5
	60 min	25	28.1
	75 min	1	1.1
Type of setting ^(c)^	School	46	50.5
	Car traffic-free environment	44	48.4
	Home	40	44.0
	Clinic	30	33.0
	Bike path	22	24.2
	Velodrome	2	2.2
	Trail (e.g., mountain bike track)	2	2.2

^(a)^ n = 90 respondents; ^(b)^ n = 89 respondents; ^(c)^ n = 91 respondents selected an average of 2.0 responses each.

### 3.5. Barriers and Facilitators to Adaptive Cycling

**Figure 5:**
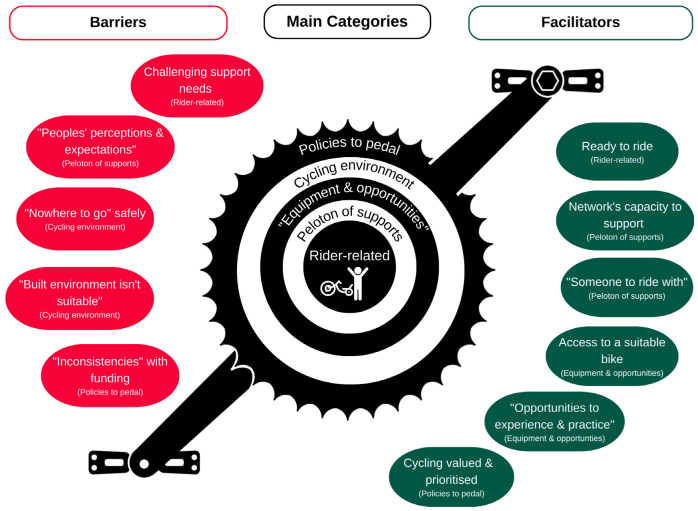
Pedal set metaphor of barriers and facilitators.

A metaphor depicting a pedal set (Figure 5) illustrates the interaction of barriers and facilitators perceived to influence a rider’s participation in adaptive cycling. At the centre are five main categories: (i) rider-related factors; (ii) ‘peloton’ of supports; (iii) equipment and opportunities; (iv) cycling environment and (v) policies to pedal. Green and red pedals representing 11 categories of facilitators and barriers were positioned alongside the central crank, enabling factors by the upper pedal and barriers around the lower pedal.

This metaphor represents the constant motion of barriers and facilitators, which were responsive to efforts or change in the rider, provider and/or environment [36]. Pedal sets can be accommodated to the rider (e.g., a shorter crank length or addition of a toe-clip), and this customizing of supports was common in textual responses, where providers were quick to highlight adaptations which could accommodate a riders’ impairments or safety needs. Of note, peloton is a cycling term for a group of riders in a race. For the purposes of this study, ‘peloton’ was used to describe the group of support personnel, both formal and informal, who act as a team around the young rider with disability.

#### 3.5.1. Rider-Related Factors

Riders were portrayed as being either “ready to ride” or having “challenging support needs”. Being “ready to ride” required rider motivation and rider attributes responsive to coaching, such as the ability to contribute to a weight-bearing transfer, “achieve the pedals motion” and “follow safety instructions”. Motivation was impacted by a reduced interest in physical activity, preference for other activities (e.g., television, gaming, team sports), disinterest in cycling with increasing age and negative adapted cycle experiences (e.g., “a crash” or feeling different on an adapted bike). A change in a riders’ priorities and a riders’ ability to keep up with others in cycling groups were raised as barriers, particularly when the performance demands (e.g., endurance or task complexity) of cycling increased.

Although there was an optimism that “there are very few health conditions or impairments that completely preclude cycling”, some support needs were challenging to accommodate “regardless of willingness and enthusiasm”. This included complex physical impairments such as movement disorders (e.g., dystonia, hypertonicity), low postural tone, established postural deformities (e.g., “fixed joint contractures” of the lower limb), limited head postural control, impaired motor function (e.g., hand strength) and anthropometric considerations (e.g., limb deficiency, short stature). Finding a comfortable and supportive assistive technology solution for these conditions was challenging (e.g., finding “suitable” lap-belts).

Safety was raised as the primary concern for young people with intellectual disability, with environmental supports such as support workers and a protected space considered as enablers to counter “poor safety awareness” and “not having executive function to manage dangers”. Providers said progressive neuromuscular conditions like Duchenne’s Muscular Dystrophy were challenging to accommodate due to the weight of the adapted bike and the adaptations available for complex physical supports. Fluctuating health conditions (e.g., refractory epilepsy) were considered “tricky because of uncertainty/inconsistency” and led some providers to choose recumbent bikes for safety reasons. “Difficulties with safe transfers on and off the bike” appeared to peak in adolescence and young adulthood and were not aided by using a hoist. Other factors impacting transfers included functional decline (e.g., post-surgery or with increasing age) and discomfort while riding (e.g., increased pain, weight-gain).

#### 3.5.2. Peloton of Supports

The peloton included formal (e.g., teacher, allied health therapist) and immediate (e.g., parent, support worker) person-based supports. Barriers and facilitators were described in relation to: (i) capacity to support, (ii) involvement of immediate supports, and (iii) “perceptions and expectations” of others.

Capacity included formal providers’ knowledge and skill, but also their availability to cohesively work with others. Having access to “knowledgeable and skilled” allied health providers and bike suppliers were reported as facilitators as they helped show young people and families that cycling “could be an option”. Training immediate caregivers to use adaptive cycling equipment and implement training plans was seen as “integral” for “real life” practice. Cohesion and communication within the peloton were thought to lead to better bike choice, the attainment of shared goals and increased practice. Knowledge gaps of the peloton in assistive technology options, accessible cycling routes and local cycling initiatives were reported as barriers. Several providers highlighted issues with generic assistive technology prescription, the poor monitoring of a rider’s musculoskeletal or developmental growth and limited planning for community cycling (e.g., fit for purpose relative to local terrains), all of which could impact bike use.

“Someone to ride with” often relied on immediate caregivers (e.g., parents, teacher aide) and sometimes peers and grandparents. A family’s involvement in adaptive cycling goals was the most cited facilitator for successful participation. Other enablers identified were cycling as a family priority, cycling practice as part of daily routines, and retaining connection to the prescribing team. Ultimately, long-term participation was perceived to be more likely with an actively engaged family or cycling companion.

Attitudes which placed cycling in the “too hard basket” was reported as a barrier. There was a perception in which some families found cycling goals difficult due to logistical challenges (time demand, practical transfers) and administrative burdens. Providers “limited” cycling practice to the “home and surrounds…” as they anticipate that cycling would be unsafe in the open community, particularly near car or bike traffic.

#### 3.5.3. Equipment and Opportunities

Cycling interventions included assistive technology (e.g., adapted cycle) and cycling “opportunities”, such as formal programs or informal riding with caregivers. Three sub-categories related to adapted cycle access, bike design features and cycling opportunities (informal and formal).

Access to a “suitable” well-fitting adapted bike or trike was considered essential and was possible by either owning or loaning the assistive technology device. Bike access was described as being impeded by maintenance issues and resourcing (e.g., limited loan-pool of adult-sized bikes) and was reliant on a thorough assessment and assistive technology prescription process. Facilitators for prescription included allied health-led assessment, “sustained trials”, exploring “different options” and having “the family and school [as] part of the trial process”.

Adapted cycle design needed to “meet the child’s unique needs” but be user-friendly for the peloton to help “manoeuvre” the bikes during cycling practice. The provision of bike frames needed to foster function and over-provision of “universal” upright-style tricycle frames, risked “abandonment” when they became “tippy” for older children or “too cumbersome” for carers supporting bike use. Reported facilitators included a readily manoeuvrable and transportable bike (e.g., trailer, removable or folding features) and attendant steering handles (i.e., a pole/handle mounted to the rear of the bike with connection to the steering). The steering handle was an enabler for new riders or people with cognitive impairment, offering an environmental safety control, but an occupational hazard for support staff pushing “heavier” riders.

Providers valued informal carer-led “opportunities for regular, fun rides with associated social interactions”. Programs in special school, which were either curriculum-led (e.g., bike education) or experiential play/practice (e.g., at recess), were highly valued as a safe way to “experience and practice bike riding”. Formal organized programs for developing adaptive cycle skills were seen to be lacking in both the community and education settings, with existing programs, events and school excursions geared towards two-wheel bike riders.

#### 3.5.4. Cycling Environment

The cycling environment appeared to hinge on having “somewhere to go” and built infrastructure in a rider’s “location of living” (e.g., metropolitan or regional, availability of storage, services and amenities). Providers sought quiet, enclosed spaces, away from car traffic, which were believed to be safer. Flat concrete areas were preferred, offering sufficient space for bikes with a larger turning circle. Conversely, uneven terrain, poorly maintained pathways, tight corners and excessive hills were considered barriers to adaptive cycling.

Immediate access to built facilities such as parks, trails, footpaths or gated gardens enabled meaningful practice and skill “cross-over”. Living in regional or remote jurisdictions created geographic divide, where riders, families and providers were faced with reduced access to built infrastructure and therapeutic supports.

#### 3.5.5. Policies to Pedal

Policies, particularly around public funding and resource allocation, were considered to have a substantial impact on cycling participation. Resourcing decisions led by funding bodies, policy makers and organizations (e.g., school leadership) made some providers feel powerless on impacting processes. Respondents felt adequate resourcing needed to span different stages of a cycling goal. “Inconsistencies” in funding allocation and processes led to uncertainty and additional burdens for riders, families and prescribers of adapted cycles. For example, partial funding (e.g., family co-payment), outright rejections based on some diagnoses (e.g., developmental delay and autism) and insufficient funding for support services (e.g., recruitment of a disability support worker) were reported to lead to process-related burnout, particularly for families. Favourable funding decisions were reported to have been facilitated through a shared “understanding from funding bodies that [cycling] can be used for a number of functional goals”.

Leadership teams in special schools shaped the cycling agenda and decided on cycling resource allocation and curriculum priority. Facilitators relative to school settings included the allocation of staff, designated space for practice, storage, timetabling cycling during school and budgeting for the purchase and maintenance of an adapted cycle “share pool”.

## 4. Discussion

This study provides a descriptive account of adaptive cycling practices to support young people with disability in Australia. We found that providers customize adaptive cycling interventions based on riders’ needs, goals, functional abilities and available resources. Although respondents worked with riders with different disabilities in diverse settings, the ‘essential elements’ of adaptive cycling interventions reported were goal setting, the exploration of options, bike selection, skills training and community training. Providers trained adaptive cycling skills through an “eclectic mix” of approaches with support from immediate caregivers. Adaptive cycling participation was perceived to be reliant on a supportive environment, suitable bike and safe places to practice. A gap appeared to exist when transitioning from formalized interventions to community-based active leisure participation. Providers had an expectation that parents and carers would lead community practice; however, beyond training in transfers and adapted cycle features, there was not much roadmap to guide cycling in more complex community situations.

The provision of adaptive cycling interventions may benefit from knowledge and evidence gained from research on other forms of wheeled mobility training, such as two-wheel bicycles and power mobility. Like Toovey and colleagues (2019) [13], who studied practices in bicycle training, we found variability in the approaches used for developing cycling skills. We found that providers blend theories and frameworks and ultimately aim to maximize a rider’s exposure to different bikes and task-specific practice in safe settings. Differences in approaches to two-wheel bicycle and adaptive cycling skills training are apparent. Training interventions for two-wheel bicycles skills are often marked by “high-density” intensive practice over 3–5 days within “learn to ride” camps [37] or school holiday programs [38]. Bicycle skills training often focuses on maximizing a rider’s ability to balance and pedal a moving bike, which can involve equipment modification, such as converting the bike to a balance-bike [19] or progressing through customized bike adaptations (e.g., ‘roller wheels’ in North American iCanShine camps [8]). Whereas adaptive cycle skills training was delivered over longer periods (i.e., several weeks), held in purpose-built safe locations and often integrated into a multi-modal intervention or immersive learning experience (e.g., physical activity initiative or daily routine). Equipment modification in adaptive cycling is more focused on customizing the adapted cycle for a comfortable and enjoyable riding experience and using assistive technology to facilitate function and enhance riders’ safety. Interestingly, not all participants from bicycle skills programs emerge as competent riders [3,5,7] and many require additional follow-up within their own “real-life setting”. Adapted cycles may offer some of these riders a means to participate in community cycling.

Parallels may be drawn between adaptive cycling and power mobility training as both mobility methods involve operating a moving device, often at speed, through complex situations. Researchers have identified three successive learning phases in power mobility training [39]: exploratory, operational and functional. These phases have been used to guide training, outcome measurement and decisions around assistive technology prescription [40]. The initial two phases focus on developing basic skills for moving in safe environments, whilst the functional phase is marked by operating the device in real-life situations and developing community mobility skills. The application of this learning approach may be helpful in adaptive cycle training, particularly in offering a learning pathway for young people with moderate to severe intellectual disability, who were considered by providers in this study to have “challenging needs”. For example, drawing upon Halayako’s cycling skills checklist [34], foundational adaptive cycling skills include preparation (e.g., positioning the adapted cycle for transfers, rider orthotics), transfers (e.g., step, stand, pivot or hoist), balancing (shifting weight on saddle or seat), pedalling, steering and stopping. Operational learners may begin to combine these skills or advance to new skills (e.g., gear changes) and functional riders might begin to integrate road safety skills into their repertoire. Similarly, the differentiation of training approaches from power mobility into task-orientated (e.g., skill checklists) and learning-process-orientated [41] may be useful for training adaptive cycling and bicycle riding, where, to date, cycling has primarily focused on task or skill checklists and lacked follow-up into natural settings [12].

Providers envisaged adaptive cycling interventions offering broad-ranging benefits in line with childhood disability’s F-words of fitness, fun, family, function, friends and future [42]. Participation opportunities varied but typically related to fitness or active leisure, and rarely extended to sport or transport. This finding is in line with the general population who primarily cycle for recreation [1], followed by active travel and then organized sport [14]. Like cycling research in generalized populations [18], safety concerns were reported as a barrier to cycling participation. Safety was nested in being able to ride in car traffic-free environments, but concerns extended to transfers, bike choice, people-based resourcing and transitioning riders to community-based practice. Reports of falls in previous adaptive cycling research are low [41] but come as an inherent risk in all cycling activities. The consequence of a fall can be catastrophic in terms of injury severity for some people with disability [43]; however, risk mitigation strategies can help minimize potential adverse events. These can include understanding a riders’ centre of mass and the mechanics of the bike (e.g., upright tricycles are more prone to tipping on turns/cornering), additional customizations to aid adapted cycle stability (e.g., addition of out-rigger or stabilizing wheel) and setting up the cycling environment for safety (e.g., setting a speed limit for sharp turns, a gated facility for riders who may abscond from practice). However, previous adaptive cycling interventions for beginners were typically performed at low speed (e.g. walk or jogging pace [21]), and choosing a suitable bike relative to the rider’s function and planned use may help (e.g., considering a recumbent bike frames for riders who need additional stability but plan to ride in more dynamic environments where terrain and corner changes are more widespread).

This study’s strengths included consultation with industry stakeholders in survey development, representation from diverse sectors and comprehensive reporting on completeness and missingness. Traditionally, surveys in motor skills training focus on allied health therapists’ perspectives, and this study answered the growing calls for expanded perspectives from other formal supports who lead capacity-building interventions [44]. Consultation with industry stakeholders and mixed question types aided the richness of the data, particularly the reflective accounts. Completeness may have been aided by the survey’s design, which clearly marked individual sections and meant partial responders typically completed a whole section. For those who discontinued, incompletion appeared to have been impacted by the time and the volume of survey questions. We comprehensively reported missing data, which in most cases were missing at random, except for several open-ended questions that suggested non-random missingness (e.g., respondents wrote “not applicable, no, yes, etc.”). The study’s exploratory focus and the qualitative nature of these questions meant no data imputation methods were conducted.

When interpreting the findings of this study, readers need to consider that the survey was study-specific and set within the Australian context. Although there was good distribution across each of the geographic contexts, the majority of responses were from physiotherapists and almost half of responses were from one Australian state. Several open-ended questions offered prompts, which may have been leading in nature and influenced respondents’ answers. The rationale to include prompts was influenced by stakeholder consultation and helped ensure that the survey was accessible to a broad cohort of respondents (e.g., coaches or laypeople). Other limitations included the study’s data collection during the COVID-19 pandemic where ‘typical practices’ may have differed due to a reduced service provision, the cessation of group therapy and challenges with trialling assistive technologies. To account for these differences, respondents were prompted to consider their usual practice in the two years before the global pandemic.

## 5. Conclusions

Providers in this study offered formal cycle training as a therapeutic or educational experience and customized their adaptive cycling interventions based on a riders’ needs, goals and resourcing. We identified barriers and facilitators to ongoing cycling participation, which were influenced by rider-related factors, the support peloton, cycling interventions, environment and policies. Knowledge of these factors can support the development of new interventions, which address barriers and target participation. We found a practice gap in interventions that focus on participation in community riding. The pedal set metaphor may also guide future research on adapted cycle prescription. For example, further research is needed about riders’ and providers’ perspectives of choosing cycling assistive technology relative to impairments, goals and intended use. Further research is required to ensure that formalized interventions translate into cycling for active leisure in the community.

## Figures and Tables

**Table 1:** Characteristics of survey respondents.

Demographic	Response	*n*	%
Discipline	Physiotherapist	61	57.0
	Occupational therapist	20	18.7
	Teacher	12	11.2
	Leisure or recreational therapist	5	4.7
	Teacher aide	3	2.8
	Exercise physiologist	2	1.9
	Other * provider	4	3.7
Practice setting	Community allied health (not-for-profit/charity)	38	35.5
	Allied health private practice	29	27.1
	Special education	28	26.2
	Tertiary health service	6	5.6
	Sport and recreation	3	2.8
	Other #	3	2.8
Australian state or territory	Victoria	53	49.5
	New South Wales	16	15.0
	Queensland	14	13.1
	South Australia	11	10.3
	Western Australia	8	7.5
	Tasmania	3	2.8
	Northern Territory	1	0.9
	Australian Capital Territory	1	0.9
Geographic setting (ASGS^∧^)	Metropolitan	72	67.3
	Inner regional	20	18.7
	Outer regional	14	13.1
	Remote	1	0.9
Experience in adaptive cycling (years worked)	<1 year	8	7.5
	1–5 years	32	29.9
	6–10 years	28	26.2
	11–15 years	26	24.3
	>16 years	13	12.1
Caseload age range ^(a)^	Early intervention (aged 0–6 years)	54	50.5
	Primary school (aged 5–12 years)	84	79.2
	Secondary school (aged 12–18 years)	72	67.9
	Young adults (aged 18–30 years)	37	34.9
	Adults (aged >18 years)	6	5.7
	Lifespan service (all ages)	6	5.7

All responses had n = 107 respondents with the exception of ^(a)^ caseload age range, which had n = 106 respondents, who could select multiple responses. * Other providers: Cycling coach (n = 1); cycling volunteer (n = 1); rehabilitation engineer/mechanic (n = 1); ergonomist (n = 1). # Other practice setting: Mainstream school (n = 2) and research (n = 1). ASGS^∧^ = Australian Statistical Geography Standard.

**Table 2:** Characteristics of riders with disabilities on a providers’ caseload.

Demographic	Response	*n*	%
Health condition or disability ^(a)^	Cerebral palsy	78	72.9
	Developmental delay	72	67.3
	Multiple disability	56	52.3
	Autism spectrum disorder	51	47.7
	Intellectual disability	50	46.7
	Acquired brain injury or stroke	39	36.4
	Down syndrome	36	33.6
	Sensory impairment	19	17.7
	Spinal cord injury	9	9.3
	Other *	14	13.1
Rider skill level [30] ^(b)^	Novice	95	89.6
	Advanced beginner	69	65.1
	Competent	20	18.7
	Proficient	6	5.7
	Expert	4	3.8
Riders’ access to an adapted cycle ^(c)^	Purchase/provision via NDIS^∧^	85	85.0
	School/rehabilitation loan pool	46	46.0
	Self-funded purchase	23	23.0
	Short-term loan or hire	11	11.0
	Purchase/provision via other fundings	7	7.0
	Long-term loan or hire	1	1.0
Riders’ perceived support needs for using an adapted cycle ^(d)^	Balance and stability	96	94.1
	Rider’s physical support needs	91	89.2
	Confidence	88	86.3
	Safety features	46	45.1
	Further possible modifications	45	44.1
	Other #	12	11.8
	None	2	2.0

Respondents could select multiple response options to all items above. ^(a)^ n = 107 respondents selected an average of 3.9 health conditions/disabilities each; ^(b)^ n = 106 respondents selected an average of 2.9 skill levels each; ^(c)^ n = 100 respondents selected an average of 1.7 access points each; ^(d)^ n = 102 respondents selected an average of 3.8 hypothesized rationale each. NDIS^∧^ = National Disability Insurance Scheme. * Other health condition or disability: Multiple sclerosis (n = 4); psychosocial disability (n = 4); traumatic brain injury (n = 2); neuromuscular condition (n = 1); amputation (n = 1); dyspraxia (n = 1); genetic/mitochondrial condition (n = 1). # Other support need: Requires additional assistance, e.g., via steering handle (n = 6); rider’s personal preference/goal (n = 2); assistive technology enabled inclusion (n = 2); requires tandem pilot rider (n = 1); teaching tool (n = 1).

**Table 3:** Summary of intervention descriptions for training adaptive cycling skills for beginner-level riders, mapped to the TIDieR checklist.

TIDieR Domain	No.	TIDieR Item	Finding
WHY	1.	Intervention	Training adaptive cycling skills for novice or beginner riders in car-free traffic environments.
	2.	Rationale	A means for young people with disabilities to work towards cycling goals whilst developing fitness, learning new skills and participating with others. Early skills training was expected to lead to local active leisure participation with immediate caregivers (i.e., family or a support worker).
WHAT	3.	Materials	Adapted cycle and accessories chosen for the rider’s personalized function and learning needs. Physical and instructional materials are listed in Supplementary File S5 (Figures S1 and S2).
	4.	Procedures	Six phases were found: (I) goal setting, (II) assessment, (III) bike exploration, (IV) bike selection, (V) skills training and (VI) community participation. Intervention types varied and included: Assessment of the rider and adapted cycle prescription. Experiential learning by trialling different adapted cycles. Adaptation of adapted cycles and bicycle set-up based on riders’ needs. Goal-directed training “graded” to a riders’ ability, learning style and ambitions. Structured learning through bike education in school. Task-specific training with providers or carers in ecological settings (e.g., home).
WHO	5.	Provided	Allied health professionals (i.e., physiotherapists and occupational therapists) often led the assessment phase. Therapists, teachers and other providers contributed to adaptive cycling skills training. Family members and support workers led local practice.
HOW	6.	Delivery mode	Face-to-face training delivered one-to-one or as part of a group.
WHERE	7.	Setting	“Safe” protected spaces largely away from car-traffic situations. Many providers never experienced riders cycling in traffic situations (51.6%) or participating in active travel (55.9%) or cycling sports (road-cycling: 78.5% and mountain-biking: 84.9%) as per Supplementary File S5.
WHEN AND HOW MUCH	8.	Frequency, intensity, time/duration, type	Providers offered training over several weeks, focusing on skills development rather than aerobic gains. Skills practice took 30–60 min (see Table 4 for further information).
	9.	Tailoring and modifications	Tailoring and modifications centred on changes relating to the task, adapted cycle, environment and rider.
	10.		Task modification varied the level of assistance offered or the demand (physical or cognitive) required of the rider. The adapted cycle was modified based on physical support needs and rider confidence. The practice environment was altered based on the providers’ perception of safety, rider goals and local opportunities.
EVALUATION	11.	Measuring change	Performance of the cycling activity and goal attainment [31,32] were the primary evaluative foci (see Supplementary File S5, Figure S3) [33]. This included cycle skills checklists [34], spatiotemporal parameters and levels of independence riding.
	12.		

See Supplementary File S5 for further details on resources, outcome measures and participation opportunities.

## Data Availability

The de-identified dataset, featuring the data of respondents who provided extended consent, is available on request to the corresponding author.

## Supplementary Materials

The following supporting information can be downloaded at: https://www.mdpi.com/article/10.3390/jcm12175523/s1, Supplementary File S1: Survey in full; Supplementary File S2: Reporting checklists (Table S1: The checklist for reporting results of internet e-surveys; Table S2: Checklist for the reporting of survey studies); Supplementary File S3: Survey content and development (Table S3: Survey sections and questions types; Table S4: Guidance for reporting the involvement of patients and the public—short form; Supplementary File S4: Pre-information and advertisement material (plain language summary; email invitation; social media invitation; poster; visual on different types of adapted cycles); Supplementary File S5: Resources, outcome measures and participation opportunities (Figure S1: Physical resources in adaptive cycling interventions; Figure S2: Informational resources in adaptive cycling interventions; Figure S3: Outcome measurements in adaptive cycling interventions; Table S5: Providers’ rating of importance for hypothesized factors to start cycling (i.e., providers’ goal); Table S6: Providers’ rating of importance for hypothesized factors that lead a rider to stop adaptive cycling; Figure S4: Frequency of perceived participation in different settings; Figure S5: Frequency of perceived participation in different activities) and Supplementary File S6: Barriers and Facilitators’ Table (Figure S6: Overview of qualitative content analysis audit trail; Table S7: Coding tree for barriers and facilitators).
