# Supplementary material for: Exploring Adaptive Cycling Interventions for Young People with Disability: An Online Survey of Providers in Australia
Source: J Clin Med. 2023 Aug 25;12(17):5523. doi: 10.3390/jcm12175523 (PMC10488225; doi:10.3390/jcm12175523)
Supplement: Supplementary file 1 [file jcm-12-05523-s001.zip › Supplemental file S1_Survey in full_final.pdf]

# Supporting cycling for children and young people (2-30 years old) with disability (Part I): a current practice survey of adapted cycling interventions.

Instructions:

The survey contains both multiple-choice (tick box) and open-response questions (enter text).

When answering these questions, please select the response that is most representative of the riders, caseload or group that you work with.

This survey will take 15-20 minutes to complete.

**Purpose of this Research: The purpose of this study is to describe current practices which support children and young people with disability to use an adapted bike or trike.**

**Please take a moment to familiarise yourself with the Plain Language Statement, which can be found in the link attached below.**

Plain Language summary

[Attachment: "CPS_20210222_Appendix M PLS Current Practice Survey_V002 UoM Ethics Approved.docx"]

For this survey, we are interested in the experiences of individuals who are:

A) Registered or trained, in Australia, and working or volunteering as:

Occupational therapists (OT) or physiotherapists registered with AHPRA (Australian Health Practitioner Regulation Agency). Teachers with an accredited teaching qualification and registered with their respective state regulatory board. Cycling coaches who have ≥AusCycling Introductory Skills Instructor qualification. Triathlon Australia coaches who have ≥Try Stars (Foundation) qualification. Sports and recreation practitioners (professional or volunteer) who lead the delivery of cycling programs. AND

B) have previous experience in providing cycling interventions or training for children and young people with disability (aged 2-30 years) who use an adapted bike or trike in Australian settings.

AND

C) Aged 18 years or older.

AND

D) Can complete a survey in English.

If you'd like to check out what an adapted bike or trike looks like, please click on the link below.

[Attachment: "CPS_20210222_Appendix B Photos Adapted Bikes and Trikes V002 UoM Ethics Approved.docx"]

| | |
|---|---|
| Do you satisfy all of the criteria described above? | ◯ Yes<br>◯ No |
| Have you completed the online training program CHAIN, which is being piloted for practitioners who work in Victoria (only) from mid April 2021? | ◯ Yes<br>◯ No<br>◯ Partial completion<br>◯ Not applicable for me |

If you are considering undertaking CHAIN and are eligible to do so (Victorian based), we ask you to consider (where possible), to complete this current practice survey first.

University of Melbourne Consent Form

1. I consent to participate in this project, the details of which have been explained to me, and I have been provided with a written plain language statement to keep.

2. I understand that the purpose of this research is to describe the current practices of occupational therapists (OT), physiotherapists, teachers, cycling coaches and sports and recreational practitioners in providing cycling interventions for children and young people (2-30 years old) with disability who use an adapted bike or trike.

3. I understand that my participation in this project is for research purposes only.

4. I acknowledge that the possible effects of participating in this research project have been explained to my satisfaction.

5. In this project I will be required to complete an online survey which takes approximately 15-20 minutes.

6. I understand that my participation is voluntary and that I am free to withdraw from this project anytime without explanation or prejudice and to withdraw any unprocessed data that I have provided.

7. I understand that the data from this research will be stored at the University of Melbourne and will be destroyed 5 years after final publication.

8. I understand that extended consent will allow for the data I provide to be shared with other ethically approved research projects in the areas of cycling, disability, assistive technology. I understand that my personal identifiable information (e.g. e-mail address) will not be shared and will continue to be stored separately and safely in the University of Melbourne’s secure server.

9. I have been informed that the confidentiality of the information I provide will be safeguarded subject to any legal requirements; my data will be password protected and accessible only by the named researchers.

10. I understand that after I tick the, “yes” I consent tickbox, on this online consent form, I am telling the research team that I:

a) understand what I have read b) I had a chance to ask questions and received satisfactory answers c) I consent to taking part in the project.

[Attachment: "CPS_20210222_Appendix N Consent Form Current Practice Survey V002 UoM Ethics Approved.docx"]

| | |
|---|---|
| Do you consent to the terms outlined in the study consent form? | ◯ Yes<br>◯ No |
| Do you provide 'extended consent', so we can share information from this study with other ethically approved research projects in related areas (e.g. disability, cycling and assistive technology)? | ◯ Yes<br>◯ No |

**Thank you for your time and consent for taking part in this survey.**

**We hope that your shared experiences in adapted cycling can help support further cycling opportunities for children and young people across Australia.**

**There are 5 sections to this survey:**

**Section 1: About you (demographics)**

**Section 2: Networks and roles**

**Section 3: Getting riders involved in cycling**

**Section 4: Working with rider's needs, abilities and goals**

**Section 5: Cycling opportunities**

Common Terminology:

"Rider with disability" refers to any child or young person with disability, aged 2-30 years of age, who has cycling goals or participates in cycling. This term has been used in the interests of inclusivity and reflects the rider's interest and participation in cycling rather than their skill level.

An "adapted bike or trike" refers to a customized, modified, supportive or specialised bicycle or tricycle that has specific features in place to cater for the needs of a person with disability.

The word adapted has been used to avoid confusion, and is most consistent with other research in this area. For the purposes of this research, we are primarily referring to bikes that are not a standard 2-wheel bicycle (e.g. BMX, standard road bike) or a static exercise bike.

"Cycling intervention" refers to any of the activities, tasks or interventions that enable participation in cycling for riders with disability.

For example, this may include, but not limited to; prescription of an adapted bike through the National Disability Insurance Scheme (NDIS), coaching cycling at sports club or teaching road skills at a Bike Education day at school and community traffic school.

**Section 1: About You (Demographics)**

**In this section we welcome you to share a little bit more about yourself and your experience supporting adapted cycle interventions for young riders with disability.**

| | |
|---|---|
| Where do you live? | ◯ Australian Capital Territory<br>◯ New South Wales<br>◯ Northern Territory<br>◯ Queensland<br>◯ South Australia<br>◯ Tasmania<br>◯ Victoria<br>◯ Western Australia |
| What state/territory does most of your work take place in? | ◯ Australian Capital Territory<br>◯ New South Wales<br>◯ Northern Territory<br>◯ Queensland<br>◯ South Australia<br>◯ Tasmania<br>◯ Victoria<br>◯ Western Australia |
| What geographical context best describes the setting where you work? https://www.abs.gov.au/websitedbs/d3310114.nsf/home/remoteness+structure | ◯ Major Metropolitan City<br>◯ Inner Regional<br>◯ Outer Regional<br>◯ Remote<br>◯ Very Remote<br>(Please see attached link if unsure) |
| When working with riders with disability, what is your job title or profession? | ☐ Allied Health Professional<br>☐ Cycle or Triathlon Coach<br>☐ Sports and Recreation Practitioner<br>☐ Teacher<br>☐ Other (Please Describe) |
| Allied Health Professional | ◯ Occupational Therapist<br>◯ Physiotherapist |
| Cycle or Triathlon Coach | ◯ AusCycling (previously Cycling Australia) Registered Coach (or relevant state/territory body)<br>◯ Triathlon Australia Accredited Coach |
| AusCycling (previously Cycling Australia) Coach Qualification | ◯ Introductory Skills Instructor<br>◯ Community Club Coach<br>◯ Level 1 Coach<br>◯ Level 2 Coach<br>◯ Level 3 Coach<br>◯ None of the above |

| | |
|---|---|
| Triathlon Australia Coach Qualification | ◯ Try Stars (Foundation)<br>◯ Tri Active (Foundation)<br>◯ Foundation Coach<br>◯ Development Coach<br>◯ Performance Coach<br>◯ High Performance Coach<br>◯ None of the above |
| Teacher | ◯ Health and Physical Education<br>◯ Special Education Teacher<br>◯ Other (Please Describe) |
| Teacher (Other: Please Describe) | __________________________________ |
| Other job title/profession (Please Describe) | ____________________________ |
| What best describes your work sector? | ◯ Disability sector (Not-for-Profit, Charity, Profit-for-Purpose)<br>◯ Disability/Community (Private Practice)<br>◯ Health care sector (Rehabilitation Department)<br>◯ Health care sector (Acute Hospital)<br>◯ Education sector (Mainstream School)<br>◯ Education sector (Specialist School or Specialist Development School)<br>◯ Sports and recreation sector<br>◯ Other (Please Describe)<br>((Please select your primary setting where >50% of your work takes place)) |
| Other Work Sector (Please Describe) | __________________________________ |
| Over the past 2 years, approximately what percentage of your work was related to supporting cycling opportunities for riders with a disability who use an adapted bike or trike? | ◯ 0-10%<br>◯ 10-25%<br>◯ 25-50%<br>◯ 50-75%<br>◯ 75-100%<br>((Please account for a typical period over the past 2 years)) |
| How many years have you worked with riders with disability who use adapted bikes or trikes? | ◯ 0-1 year<br>◯ 1- 5 years<br>◯ 6-10 years<br>◯ 11-15 years<br>◯ >16 years |
| How have you acquired your skills to work with riders who use adapted bikes and trikes?<br><br>(e.g. on the job, a specific course) | __________________________________<br>((Please describe briefly)) |

| | |
|---|---|
| When working with riders with disability, which disability or health condition best describes the main population you work with? | ☐ Acquired brain injury<br>☐ Autism<br>☐ Cerebral palsy<br>☐ Hearing impairment<br>☐ Intellectual disability<br>☐ Developmental delay<br>☐ Global developmental delay<br>☐ Down syndrome<br>☐ Multiple Sclerosis<br>☐ Psychosocial disability<br>☐ Spinal cord injury<br>☐ Stroke<br>☐ Vision impairment<br>☐ Multiple Disability (i.e. combination of ≥2 intellectual, psychosocial, sensory/speech, physical/diverse disabilities)<br>☐ Other (please describe)<br>((Please select up to three which are most representative of your practice)) |
| Other Disability or Health Condition (Please Describe) | ______________________ |
| What age group do you work with? | ☐ Early Intervention (0-6 year old)<br>☐ Kinder (3-5 year old)<br>☐ Primary School (5-12 year old)<br>☐ Secondary School (12-18 year old)<br>☐ Young Adult Service (18-30 year old)<br>☐ Other (please describe) |
| Other Age Range (Please Describe) | ______________________ |
| When working with riders who use an adapted bike or trike, what skill level do you typically work with? | ☐ Novice/Absolute Beginner (e.g. learn to ride program)<br>☐ Advanced Beginner (e.g. basic cycling skills in safe environments)<br>☐ Competent (e.g. refining advanced cycling skills [e.g. gears] in age-appropriate contexts)<br>☐ Proficient (becoming automatic with cycling skills in a range of settings)<br>☐ Expert (mastered cycling in many contexts and could compete at an elite level) |

## Section 2: Networks and roles

**This information helps us to further understand more about the support network around young riders and some of the support roles you take on.**

Aside from the rider, who do you work alongside when you provide cycling intervention?

- ☐ Rider's immediate supports (e.g. parent/guardian)
- ☐ Health Professionals
- ☐ Educators (e.g. teacher)
- ☐ Support Worker (e.g. disability support worker)
- ☐ Assistive Technology Specialists (e.g. bike supplier)
- ☐ Community Cycling or Sports Bodies (e.g. coaches)
- ☐ NDIS Representatives (e.g. Local Area Coordinator)
- ☐ Other (Please Describe)

((Please select all that apply to you))

---

Other Support Network (Please Describe)

__________________________________

---

Does your role in cycling intervention involve supervising or mentoring anyone else?

e.g. coaching a parent to deliver a home program, supervising a therapy assistant.

- ◯ Yes
- ◯ No

---

Please list who you support or mentor to co-deliver cycling interventions.

__________________________________

---

What cycling programs or activities are you involved in?

- ☐ Cycle skills training
- ☐ Cycle coaching in community sports
- ☐ Bike education for another sector (e.g. Ride2School, Active Transport Initiative)
- ☐ Assistive technology prescription
- ☐ Cycle holidays or organised excursion (e.g. camp, outdoor education)
- ☐ Paralympic classifier for cycling
- ☐ Other (Please Describe)

---

Other cycling activity/program (please describe)

__________________________________

| | |
|---|---|
| What cycling interventions are you involved in? | ☐ Assessment of the rider's function (e.g. posture, support needs)<br>☐ Fitting, modification, prescription of the adapted bike or trike<br>☐ Cycle Skills Training (e.g. steering, braking)<br>☐ Transfer Practice (i.e. getting on/off their bike or trike safely)<br>☐ Road safety skills training with the rider or their support person<br>☐ Teaching bike maintenance skills (e.g. changing tyre) with rider/family<br>☐ Participation-focused interventions (e.g. using motivational interviewing or solution-focused coaching)<br>☐ None of the above<br>☐ Other (Please describe)<br>((Please select all that apply to you)) |
| Other cycling intervention (Please Describe) | ______________________________ |
| In the past, why have you suggested an adapted bike or trike for some riders with disability? | ______________________________<br>((Please take a moment to reflect, describe and share your experiences)) |
| Which of the following factors, if any, were important in your reasons for suggesting an adapted bike or trike? | ☐ More balance and stability than other cycling options<br>☐ Allowed rider to gain more confidence to cycle<br>☐ Catered for rider's support needs (e.g. postural supports such as back-rest)<br>☐ Further modification possible with change in ability (e.g. power add-on)<br>☐ Additional safety features (e.g. side-by-side bike)<br>☐ Other (Please Describe)<br>☐ None of the above |
| Other factor (please describe) | ______________________________ |
| What goal(s) do you typically envisage achieving through the suggestion of an adapted bike or trike? | ______________________________<br>((Please list or use dot points)) |

**On a scale from 1-5, where 1 is 'not important at all' and 5 represents 'extremely important', how important were the following goals which may have led you to suggesting an adapted bike or trike?**

| | 1: Not important at all | | 3: Neutral | | 5: Extremely important |
|---|---|---|---|---|---|
| A way to work towards riding a standard 2-wheel bike | ○ | ○ | ○ | ○ | ○ |
| A way to work on rehabilitation goals (e.g. increasing movement after surgery) | ○ | ○ | ○ | ○ | ○ |
| A way to work on life skills (e.g. safety awareness, social skills) | ○ | ○ | ○ | ○ | ○ |
| A way for the rider to play in safe or protected environments | ○ | ○ | ○ | ○ | ○ |
| A way for the rider to get around the community (e.g. school commute) | ○ | ○ | ○ | ○ | ○ |
| A way for the rider to access sport | ○ | ○ | ○ | ○ | ○ |
| A way for the rider to develop strength or fitness | ○ | ○ | ○ | ○ | ○ |

**Section 3: Getting riders involved in cycling**

**In this section we ask you to consider how children and young people get involved in cycling.**

**We are also interested in the contexts that young people with disability participate in cycling with an adapted bike or trike.**

In your experience, what age do children and young people with disability typically start cycling with an adapted bike or trike?

__________________________
((Please specify an age in years))

In your experience, who typically leads the decision to choose cycling as an activity or goal for a child or young person with disability?

- ◯ Child or young person themselves
- ◯ Parent or family member
- ◯ Allied health professional (e.g. OT or physiotherapist)
- ◯ School teacher or educator
- ◯ Sports and recreation practitioner
- ◯ Cycling Coach
- ◯ Shared decision with the child, family and wider team
- ◯ Other (Please describe)

Other Leads Choice (Please Describe)

__________________________________

How do the riders you work with most commonly access an adapted bike or trike?

- ☐ Self-funded
- ☐ Prescribed and provided through NDIS funding
- ☐ Shares a bike or trike with others (e.g. share pool of equipment in specialist school)
- ☐ Loans a bike or trike from a voluntary body (temporary)
- ☐ Rents a bike or trike from a supplier or hire company (longer term loan)
- ☐ Other please explain

((You can select up to three))

Other Access to Adapted Bike or Trike (Please Describe)

__________________________________

| | |
|---|---|
| In cases where a child or young person doesn't wish to choose cycling as an activity within your program or has difficulty accessing a suitable bike, what other active play/sport equipment/mobility equipment, if any, is considered in place of adapted cycling? | ☐ Walking with Posterior Walker<br>☐ Walking with Supportive gait trainer<br>☐ Supportive running bike (i.e. Race Runner)<br>☐ Riding a Scooter<br>☐ Standing Frame<br>☐ Static gym bike<br>☐ Motorised assisted movement device (e.g. assisted hand cycle)<br>☐ 2-wheel bicycling<br>☐ Unsure<br>☐ Other (Please Describe)<br>((Please select all that apply)) |
| Other Choice Active Play/Physical Activity Equipment (Please Describe) | __________________________________ |
| In your experience, do all children and young people with disability have equal opportunities to be included in cycling? | ◯ Yes<br>◯ No |
| Please briefly explain what you perceive has led to reduced inclusive cycling opportunities for riders you worked with. | __________________________________<br>(Consider listing barriers with respect to your riders) |

**In your experience, how often do children and young people with disability cycle in the following settings using their adapted bike or trike?**

| | Never | 1-2 times per year | 3-4 times per year | 1 time per month | 2-3 times per month | 1-2 times per week | 3-4 times per week | Daily |
|---|---|---|---|---|---|---|---|---|
| Home (e.g. driveway, garden, house) | ○ | ○ | ○ | ○ | ○ | ○ | ○ | ○ |
| Education setting (e.g. school) | ○ | ○ | ○ | ○ | ○ | ○ | ○ | ○ |
| Protected community areas (traffic-free). E.g. parks, local footpaths. | ○ | ○ | ○ | ○ | ○ | ○ | ○ | ○ |
| Community (including road-traffic). E.g cycle lanes. | ○ | ○ | ○ | ○ | ○ | ○ | ○ | ○ |
| Sports Club (road cycling) | ○ | ○ | ○ | ○ | ○ | ○ | ○ | ○ |
| Sports Club (mountain cycling) | ○ | ○ | ○ | ○ | ○ | ○ | ○ | ○ |
| Other (Please Describe) | ○ | ○ | ○ | ○ | ○ | ○ | ○ | ○ |

Other Setting (Please Describe) __________________________________

**In your experience, how often do riders with disability report to take part in the following activities using their adapted bike or trike?**

| | Never | 1-2 times per year | 3-4 times per year | 1 time per month | 2-3 times per month | 1-2 times per week | 3-4 times per week | Daily |
|---|---|---|---|---|---|---|---|---|
| Family cycle | ○ | ○ | ○ | ○ | ○ | ○ | ○ | ○ |
| Local cycle with support worker | ○ | ○ | ○ | ○ | ○ | ○ | ○ | ○ |
| Play with siblings or peers (e.g. recess) | ○ | ○ | ○ | ○ | ○ | ○ | ○ | ○ |
| Commute to school or work | ○ | ○ | ○ | ○ | ○ | ○ | ○ | ○ |
| Cycle with sports club | ○ | ○ | ○ | ○ | ○ | ○ | ○ | ○ |
| School physical education (PE) | ○ | ○ | ○ | ○ | ○ | ○ | ○ | ○ |

**Section 4: Working with Rider's Needs, Abilities and Goals**

**In this next section we consider the support needs of the rider.**
**This information helps us to understand more about the functional needs of riders and cycling goals and aspirations.**

In your experience, what health conditions or impairments, if any, make it difficult for a child or young person to use an adapted bike or trike?

(e.g. contracture of a certain joint, epilepsy)

__________________________________
((Please list or use dot points))

What factors contributed to successful use of an adapted bike or trike?

(e.g. rider related abilities, local opportunities, etc).

__________________________________
((Please take a moment to reflect, describe and share your experiences))

Do you evaluate change in riders' cycling abilities or goals which may result from your cycling intervention?

(e.g. marker or measure of change)

◯ Yes
◯ No

How do you evaluate change in riders' cycling abilities or goals?

__________________________________
((Please consider listing an outcome measurement, tool or strategy you use))

In your experience, what factors contribute to a child or young person with disability to stop using their adapted bike or trike or stop participating in cycling?

__________________________________
((Please take a moment to reflect, describe and share your experiences))

**On a scale from 1-5, where 1 is 'not important at all' and 5 represents 'extremely important', how important would the following contributors be for a child or young person to stop using their adapted bike or trike.**

| | 1: Not important at all | | 3: Neutral | | 5: Extremely Important |
|---|---|---|---|---|---|
| Costs of programs or cycling opportunities | ○ | ○ | ○ | ○ | ○ |
| Change in rider's function (e.g. ease of getting on/off bike, contracture) | ○ | ○ | ○ | ○ | ○ |
| Change in interests/motivation of rider | ○ | ○ | ○ | ○ | ○ |
| Transition period in rider's life (e.g. move to adult services) | ○ | ○ | ○ | ○ | ○ |
| Ability to upkeep/maintain repairs of adapted bike or trike | ○ | ○ | ○ | ○ | ○ |
| Access to skilled practitioners | ○ | ○ | ○ | ○ | ○ |
| Transporting the adapted bike or trike | ○ | ○ | ○ | ○ | ○ |
| Lack of organised cycling opportunities (e.g. inclusive clubs, programs) | ○ | ○ | ○ | ○ | ○ |
| Lack of informal cycling opportunities (e.g. support to cycle from home) | ○ | ○ | ○ | ○ | ○ |

**Section 5: Cycling Opportunities**

**Thank you for your time and valued expertise, you’re onto the final section.**

**We are interested in understanding which adapted bikes and trikes are commonly seen in practice and how you deliver your cycle intervention.**

What format do you use to deliver cycle skills training programs?

- ☐ Individual (one child at a time)
- ☐ Group-based
- ☐ Face to face (i.e. in person)
- ☐ Video consultation (e.g. TeleHealth)
- ☐ By email
- ☐ Mix of face-to-face and video conferencing (hybrid)

((Please select all that apply))

What setting(s) do you typically deliver cycle skills training?

- ☐ Clinic based
- ☐ School or education setting
- ☐ Protected community area (e.g. park, local traffic-school)
- ☐ Home environment (in/around the rider's home)
- ☐ Community cycle-paths (including traffic-zones, roadways)
- ☐ Velodrome
- ☐ Other (Please Describe)

Other setting cycle skills (Please Describe)

__________________________________

Please consider using the images of adapted bikes and trikes to assist in completing the next section.

[Attachment: "CPS_20210222_Appendix B Photos Adapted Bikes and Trikes V002 UoM Ethics Approved.docx"]

## How often, if at all, do you work with the following adapted bike or trikes?

| | Never | Sometimes | Always |
|---|---|---|---|
| Adapted Trike (Commercially Available: e.g. Gomier) | ◯ | ◯ | ◯ |
| Adapted Bike (Commercially Available: e.g. MoMo Therapy Bike) | ◯ | ◯ | ◯ |
| Customised Trike or Bike (e.g. TAD or Solve customised) | ◯ | ◯ | ◯ |
| Tandem Bike | ◯ | ◯ | ◯ |
| Side-by-side Bike | ◯ | ◯ | ◯ |
| Tapered Roller Bike (e.g. iCan Ride adapted rear wheel) | ◯ | ◯ | ◯ |
| Handcycle (upright trike set up) | ◯ | ◯ | ◯ |
| Recumbent Bike (foot or hand pedal setup) | ◯ | ◯ | ◯ |
| Wheelchair Cycle (add-on) | ◯ | ◯ | ◯ |
| Trailer Bike | ◯ | ◯ | ◯ |
| E-Bike or Trike (electric/power add-on) | ◯ | ◯ | ◯ |
| Company Cycle (e.g. Wheelchair Cargo Mount) | ◯ | ◯ | ◯ |
| Prone Recumbent Bike | ◯ | ◯ | ◯ |
| Other (Please Describe) | ◯ | ◯ | ◯ |

Other adapted bike or trike (Please describe)

__________________________________

How long does a typical cycle skills training session last for?

◯ < 20 minutes
◯ 30 minutes
◯ 45 minutes
◯ 60 minutes
◯ 75 minutes
◯ Other (Please describe)

Other time (Please Describe)

__________________________________
((Please include time in minutes: e.g. 90 is 1.5 hours))

| | |
|---|---|
| What exercise intensity, if any, do you aim for riders to work at? | ◯ Focus is primarily on skills development<br>◯ Unsure<br>◯ Low intensity (Easy exercise, no effort, comfortable)<br>◯ Moderate intensity (light exercise, little strain, could go on for hours)<br>◯ Moderate to vigorous intensity (Getting quite hard, can hear heavy breathing, rosy cheeked, lightly sweating)<br>◯ Vigorous intensity (Very hard exercise, out of breath or can just manage 1-2 words) |
| How often are your cycle skills training sessions? | ☐ A once off session<br>☐ Intensive block: 3-5 days over 2 weeks (e.g. during school holidays)<br>☐ Once weekly for a number of weeks (e.g. weekly over a school term)<br>☐ As requested by the rider/family<br>☐ Other (Please Describe) |
| Other Frequency (Please Describe) | __________________________________ |
| Aside from the adapted bike or trike, what physical equipment do you need to run your cycling program? | __________________________________<br>((Please list or use dot points)) |
| What informational material or educational resources, if any, do you use during your cycling program? | __________________________________<br>((Please list or use dot points)) |
| Is there a motor theory or teaching/coaching approach that informs your practice when delivering cycle skills training?<br><br>(e.g. cognitive orientated approach, task-specific training, discrete trial training) | ◯ Yes<br>◯ No<br>◯ Unsure |
| Please list what approach(es) or theory informs your practice.<br><br>(e.g. CO-OP approach, structured teaching, context therapy) | __________________________________<br>((Please take a moment to reflect, describe and share your experiences)) |
| What were the most important factors for you in choosing this approach or theory? | __________________________________ |
| Is there anything else you'd like to add or share that you feel could help others to support riders with disability who use adapted bikes or trikes? | __________________________________<br>((Open Response)) |

| | |
|---|---|
| End of Survey<br><br>Thank you for participating in our survey.<br>Your feedback helps us to champion cycling and support more opportunities for young riders and their families to cycle.<br><br>Would you like to sign up to receive a copy of the study's results? | ◯ Yes<br>◯ No |
| Please enter the email address which you would like to be contacted on to receive results. | __________________________________ |
