# Supplementary material for: Exploring Adaptive Cycling Interventions for Young People with Disability: An Online Survey of Providers in Australia
Source: J Clin Med. 2023 Aug 25;12(17):5523. doi: 10.3390/jcm12175523 (PMC10488225; doi:10.3390/jcm12175523)
Supplement: Supplementary file 1 [file jcm-12-05523-s001.zip › Supplemental file S2_Reporting checklists_publish.pdf]

Supplemental File S2: Reporting checklists

Table S1: The Checklist for Reporting Results of Internet E-Surveys (CHERRIES) [25]

Item Category	Checklist item	Page #	Description
**Design**	Describe survey design	1, 2, Supp. 2	A cross-sectional online survey hosted on REDCap and accessed by a convenience sample of allied health providers, teachers and cycling instructors with experience of leading adaptive cycling interventions in Australia. The survey was developed as a study-specific survey for self-administration by respondents on a once-off basis.
**Institutional Review Board (IRB) approval and informed consent process**	IRB approval	2	Ethics approval from the University of Melbourne's Human Ethics Research Committee (reference: 20449, approved 10/03/2021).
	Informed consent	2, Supp. 4	Prior to the provision of informed consent, potential participants could view and download a detailed plain language summary (PLS). The PLS provided information on survey length, study aims/rationale, data storage, and investigator contact details. Informed consent was obtained electronically through tick-boxes within the survey's opening page.
	Data protection	Supp. 4	Data was stored in a password-protected, secure server in the University of Melbourne. All data was de-identified for analysis and reporting, and re-identifiable data was stored separately in "raw data" file. The dataset is only accessible to members of the research team (JJC, RT, AJS) and will be stored for 5 years, after which it will be securely destroyed.
**Development and pre-testing**	Survey development and piloting	3, 4 Supp. 3	Piloting focused on content, usability, and time to complete. The pilot sample was representative of our desired study population.
**Recruitment process and access to the survey**	Open versus closed survey	2, Supp. 1, 4	This was an open survey accessed via the recruitment material's weblink or QR code. An eligibility screen and the provision of project information (i.e. PLS) preceded consent procedures.
	Contact mode	3 Supp. 3	Mixed recruitment methods were used including targeted advertisement and snowballing.
	Survey advertisement	Supp. 4	Advertisement materials are detailed in Supplemental File 4. Materials were shared by email or social media.
**Survey administration**	Web/e-mail	2 Supp. 1	A web-based survey managed using REDCap's web application [24].

	Context	3, 15, 16 Supp. 3	Study information (Supplemental file 4) was shared with targeted organizations via email and social media (Facebook, LinkedIn and Twitter). The social media channels included professional practice groups/communities of practice where participants can seek information from peer networks, share information and research studies. Cycling, adapted physical activity and current practices feature regularly within the social media groups.
	Mandatory/ voluntary	Supp. 4	Participation was voluntary.
	Incentives	3 Supp. 4	There were no incentives for taking part. Respondents could choose to provide their email address to receive a copy of the study's results.
	Time/ date	4	The study was open for an eight-month period (March 31st- November 19th 2021).
	Randomization of items or questionnaires	Supp. 1, 2	Survey items were not randomized.
	Adaptive questioning	3	Branching logic and adaptive questioning featured throughout the survey, and enabled respondents to tailor their survey path based on prior responses and their respective role in adaptive cycling.
	Number of items	3, Supp. 3	Eighty-two questions were presented to all respondents (Supplemental file 3). The overall number of items was variable and determined by the respondent's unique survey path (i.e. branching logic, adaptive questioning and responses to optional questions).
	Number of screens or survey pages	Supp. 2	Respondents could view their progression over 12 pages. Each of the five sections were numbered, however individual question items were not numbered.
	Completeness check	4, 16	The completeness check was completed after survey responses were submitted by highlighting completeness of mandatory items and taking account of a participant's survey pathway.
	Review step	Supp. 2	Respondents could review and change their responses (e.g. through a "reset" button or "previous page").
**Response rates**	Unique site visitor	2, Supp. 4	As respondents could choose to remain fully anonymous, this study did not capture each unique visitor's IP address.

	View rate	2, Supp. 4	Not applicable (unique site visitor data not collected).
	Participation rate	2, Supp. 4	As above.
	Completion rate	3-5	The completion rate was 84.1% (i.e. n=90 respondents completed section 5, while 17 respondents provided consent but were partial responders). The completeness check and missing data is reported in Figure 1.
**Preventing multiple entries from the same individual**	Cookies used	N/A	Cookies were not used during this study.
	Internet protocol (IP) address check	2, Supp. 4	IP was not checked during this study as participants could chose to remain fully anonymous.
	Log file analysis	3, 4, 15, 16	A partial check for multiple entries was possible through email identifier (provided by n=61 respondents), timestamp and work/location based demographics.
	Registration	N/A	Not applicable.
**Analysis**	Handling of partially complete surveys	3-4	Due to the exploratory nature of the study, all responses, including partial responses were reported.
	Surveys with an atypical timestamp	3, 4, 15, 16	There were no atypical timestamps identified. There was no survey time-frame cut-off point, the median time spent on REDCap web-application was 25.0 minutes (range 2.0-183.0 minutes).
	Statistical correction	N/A	Not applicable.

Footer: Supp.= Supplemental; N/A= Not applicable.

Supplemental File S2: Reporting checklists

Table S2: Checklist for Reporting Of Survey Studies (CROSS) by Sharma et al (2021) [26].

Section/topic	Item	Item description	Reported on page #
**Title and abstract**			
Title and abstract	1a	State the word "survey" along with a commonly used term in title or abstract to introduce the study's design.	1
	1b	Provide an informative summary in the abstract, covering background, objectives, methods, findings/results, interpretation/discussion, and conclusions.	1
**Introduction**			
Background	2	Provide a background about the rationale of study, what has been previously done, and why this survey is needed.	1-2
Purpose/aim	3	Identify specific purposes, aims, goals, or objectives of the study.	2
**Methods**			
Study design	4	Specify the study design in the methods section with a commonly used term (e.g., cross-sectional or longitudinal).	2-3<br>Study-specific survey with mixed question types (close-ended, open-ended descriptive and open-ended reflective). Close-ended questions included checkboxes (e.g. single or multiple choice) and scales (e.g. frequency, level of importance). Open-ended descriptive asked respondents to list or name factors. Open-ended 'reflective' questions encouraged respondents to "take a moment to reflect, describe and share your experiences".

	5a	Describe the questionnaire (e.g., number of sections, number of questions, number and names of instruments used).	3,<br>Supp. 3
	5b	Describe all questionnaire instruments that were used in the survey to measure particular concepts. Report target population, reported validity and reliability information, scoring/classification procedure, and reference links (if any).	3,<br>Supp. 3<br>Australian Statistical Geography Standard (ASGS) for geographic setting and Dreyfus model [30]/AUS Physical Literacy Framework for rider skill level.
Data collection methods	5c	Provide information on pretesting of the questionnaire, if performed (in the article or in an online supplement). Report the method of pretesting, number of times questionnaire was pre-tested, number and demographics of participants used for pretesting, and the level of similarity of demographics between pre-testing participants and sample population.	3<br>Supp. 3.
	5d	Questionnaire if possible, should be fully provided (in the article, or as appendices or as an online supplement).	Supp. 1
	6a	Describe the study population (i.e., background, locations, eligibility criteria for participant inclusion in survey, exclusion criteria).	3
Sample characteristics	6b	Describe the sampling techniques used (e.g., single stage or multistage sampling, simple random sampling, stratified sampling, cluster sampling, convenience sampling). Specify the locations of sample participants whenever clustered sampling was applied.	3

	6c	Provide information on sample size, along with details of sample size calculation.	3 (anticipated recruitment size)<br>Due to the exploratory nature of this study, a sample size calculation was not formally performed.
	6d	Describe how representative the sample is of the study population (or target population if possible), particularly for population-based surveys.	Consultation with stakeholders (Supp. 3) aided the choice of the target population. Pre-testing involved a pilot group who represented the desired provider population.
Survey administration	7a	Provide information on modes of questionnaire administration, including the type and number of contacts, the location where the survey was conducted (e.g., outpatient room or by use of online tools, such as SurveyMonkey).	2-3<br>Supp. 2 (above)
	7b	Provide information of survey's time frame, such as periods of recruitment, exposure, and follow-up days.	4<br>Supp. 2 (above)
	7c	Provide information on the entry process:<br>–>For web-based surveys, provide approaches to prevent "multiple participation" of participants.	3
Study preparation	8	Describe any preparation process before conducting the survey (e.g., interviewers' training process, advertising the survey).	Supp. 4<br>Respondents were provided with a list of common terminology in adaptive cycling, visuals of common adapted cycle options and advertisement material.
Ethical considerations	9a	Provide information on ethical approval for the survey if obtained, including informed consent, institutional review board [IRB] approval, Helsinki	2,<br>Supp. 4

		declaration, and good clinical practice [GCP] declaration (as appropriate).	Respondents could opt to provide extended consent so their de-identified data could be shared with researchers in related fields/methodology.
	9b	Provide information about survey anonymity and confidentiality and describe what mechanisms were used to protect unauthorized access.	2<br>Supp. 4<br>The survey was developed, hosted and managed through REDCap, which is a secure web-based application for surveys and databases. Datasheets were de-identified prior to analysis, with removal of any named providers, programs, services or locations. A data dictionary was used to track re-identifiable terminology.
Statistical analysis	10a	Describe statistical methods and analytical approach. Report the statistical software that was used for data analysis.	4, 5<br>Supp.6
	10b	Report any modification of variables used in the analysis, along with reference (if available).	4,<br>Supp. 5
	10c	Report details about how missing data was handled. Include rate of missing items, missing data mechanism (i.e., missing completely at random [MCAR], missing at random [MAR] or missing not at random [MNAR]) and methods used to deal with missing data (e.g., multiple imputation).	4, 15, 16<br>Cases of missingness were noted in the footnote of tables and figures. Completeness and missingness was reported for mandatory responses (Figure 1 Page 4). Data was reported as missing completely at random, with the exceptions of cases where a respondent cited "not applicable or no" (missing not at random).
	10d	State how non-response error was addressed.	N/A.
	10e	For longitudinal surveys, state how loss to follow-up was addressed.	N/A

	10f	Indicate whether any methods such as weighting of items or propensity scores have been used to adjust for non-representativeness of the sample.	N/A
	10g	Describe any sensitivity analysis conducted.	N/A
**Results**			
Respondent characteristics	11a	Report numbers of individuals at each stage of the study. Consider using a flow diagram, if possible.	4
	11b	Provide reasons for non-participation at each stage, if possible.	4, 15, 16
	11c	Report response rate, present the definition of response rate or the formula used to calculate response rate.	Due to the mixed methods of recruitment (targeted and snowball) a definitive response rate could not be calculated.
	11d	Provide information to define how unique visitors are determined. Report number of unique visitors along with relevant proportions (e.g., view proportion, participation proportion, completion proportion).	Unique visitors were unable to be determined as we did not track IP- see Supp. 2 above.<br><br>4-5
Descriptive results	12	Provide characteristics of study participants, as well as information on potential confounders and assessed outcomes.	4-8
Main findings	13a	Give unadjusted estimates and, if applicable, confounder-adjusted estimates along with 95% confidence intervals and p-values.	N/A.<br>A Pearson chi test found no statistical differences (0.6) when cross-checking survey completion rate between the 3 primary response groups (i.e. physiotherapists, occupational therapists and other).
	13b	For multivariable analysis, provide information on the model building process, model fit statistics, and model assumptions (as appropriate).	N/A

	13c	Provide details about any sensitivity analysis performed. If there are considerable amount of missing data, report sensitivity analyses comparing the results of complete cases with that of the imputed dataset (if possible).	N/A
**Discussion**			
Limitations	14	Discuss the limitations of the study, considering sources of potential biases and imprecisions, such as non-representativeness of sample, study design, important uncontrolled confounders.	15, 16
Interpretations	15	Give a cautious overall interpretation of results, based on potential biases and imprecisions and suggest areas for future research.	14-16
Generalizability	16	Discuss the external validity of the results.	14-16
**Other sections**			
Role of funding source	17	State whether any funding organization has had any roles in the survey's design, implementation, and analysis.	16
Conflict of interest	18	Declare any potential conflict of interest.	17
Acknowledgements	19	Provide names of organizations/persons that are acknowledged along with their contribution to the research.	17
