# Supplementary material for: Exploring Adaptive Cycling Interventions for Young People with Disability: An Online Survey of Providers in Australia
Source: J Clin Med. 2023 Aug 25;12(17):5523. doi: 10.3390/jcm12175523 (PMC10488225; doi:10.3390/jcm12175523)
Supplement: Supplementary file 1 [file jcm-12-05523-s001.zip › Supplemental file S3_Content and development GRIPP2-SF_final.pdf]

Supplemental File S3: Survey content and development (GRIPP2-SF)

Table S3: Survey sections and breakdown of question types

	Section	Question Types				Instrument or scale
		CE*	OE# (list)	OE (reflect)	Total	
1.	About you (provider demographics)	11	1	0	12	Provider geographical context: Australian Statistical Geography Standard, accessed online (05/06/2023) via: https://www.abs.gov.au/statistics/statistical-geography/australian-statistical-geography-standard-asgs<br><br>Rider disability: National Disability Insurance Scheme primary disability types, accessed online (05/06/2023) via: https://www.ndis.gov.au/applying-access-ndis/how-apply/information-support-your-request/types-disability-evidence<br><br>Rider skill: Dreyfus model [30] and AUS Sports Physical literacy framework, accessed online (05/06/2023) via: https://www.sportaus.gov.au/physical_literacy
2.	Networks and roles	12	1	2	15	5-point Likert importance
3.	Getting riders involved in cycling	17	0	1	18	8-point frequency scale
4.	Working with rider's needs, abilities, and goals	10	2	2	14	5-point Likert importance
5.	Cycling opportunities	19	3	1	23	3-point frequency scale
	Total	69	7	6	82	

Footer: CE*= Close-ended, OE#= Open ended

Supplemental File S3: Survey content and development (GRIPP2-SF)

Table S4: Public involvement in survey development and analysis reported through the Guidance for Reporting Involvement of Patients and the Public- Short Form (GRIPP2-SF) [28]

Section and topic	Item	Page
Aim	To consult [27] with cycling providers and end-users (i.e. people with lived experience of disability) during the design, distribution and analysis of this study.	
Methods	Public involvement was conducted through virtual meetings and email communication with two separate groups: a) Victorian "Cycling Alliance" b) individual stakeholders.<br><br>*Victorian "Cycling Alliance"*<br>The Victorian "Cycling Alliance" working group was formed in 2020-2021 by Dr Rachel Toovey and representatives from Disability Sport and Recreation (disability sports charity) to develop inclusive cycling resources. The 12-person group included representatives from the university sector, cycling sports (i.e. AusCycling, Bicycle Networks, Inclusive Sports Training), disability (TAD*-Solve, Special Olympics), allied health (Grow Strong Physiotherapy) and a competitive cyclist with lived experience of disability. The Cycling Alliance supported survey development through virtual discussions on cycling terminology and prospective adaptive cycling providers. Members of the Cycling Alliance assisted with recruitment of the survey through distribution among their networks and mail-lists.<br><br>*Individual stakeholder feedback*<br>Four stakeholders offered consultative input for the survey development and analysis. The stakeholders included a special education health and physical education teacher (CR), physiotherapist (AT), occupational therapist (LD) and assistive technology supplier (NW).<br>Feedback was obtained through email communication and five separate virtual meetings, each lasting 60 minutes. During the development phase, meetings focused on the survey's aim, question choice and pre-testing for relevance. The analysis phase sought email feedback on the wording and relevance of categories developed through the qualitative analysis.	
Study results	Both groups of stakeholders reviewed the early iterations of the survey and offered feedback on the survey's final design (e.g. format, item choice and industry relevance), readability and usability (e.g. preparation content on common terminology in adaptive cycling). Their feedback led to several items being revised, aided the selection of	

	terminology (e.g. adapted bike/cycle, 'riders', strengths-based perspectives), inclusion of prompts/examples and brought a participation-focus to the survey's content (environment, goals, choice). Involvement of the 'Cycling Alliance' aided recruitment to the survey, with 10 organisations involved confirming distribution amongst their networks.<br><br>Consultation with the individual stakeholders (CR, AT) during the qualitative analysis led to a face validity check of categories and improved reporting of the coding tree (Supplemental File 6) and metaphor. Stakeholders felt the results represented barriers and facilitators seen in practice. Stakeholders suggested improvements in language choice and visual presentation of "hierarchies of information" led to removal of jargon and improved labelling of categories within the pedal set metaphor.
Discussion and conclusion	Feedback from stakeholders improved the survey design, recruitment and trustworthiness of qualitative analysis.
Reflections and critical perspective	As this study aimed to gather the perspectives of cycling providers, our public involvement primarily focused on feedback from stakeholders involved in inclusive cycling opportunities. Stakeholder involvement led to a more pragmatic language choice in survey development and reporting. Although one adult with disability was involved through the 'Cycling Alliance', input from people with lived of disability was lacking. Future studies should incorporate adequate time, funding and processes (e.g. advisory panel, co-researchers) to ensure quality consumer involvement is embedded across all phases of the research process.

Footer: TAD*=Technology for Aging and Disability. Stakeholders included: CR (Clare Redenbach); AT (Abiramy Thevarajah); LD (Liz Doyle) and NW (Nick Warren).
