# Supplementary material for: Exploring Adaptive Cycling Interventions for Young People with Disability: An Online Survey of Providers in Australia
Source: J Clin Med. 2023 Aug 25;12(17):5523. doi: 10.3390/jcm12175523 (PMC10488225; doi:10.3390/jcm12175523)
Supplement: Supplementary file 1 [file jcm-12-05523-s001.zip › Supplemental file S4_Pre-information and advertisement material_final.pdf]

Supplemental File S4: Pre-information and advertisement material

Plain Language Statement:

Project: Supporting cycling for children and young people with disability: a current practice survey of adapted cycling interventions (Part 1).

Responsible Researchers: Dr Rachel Toovey
Tel: +613 8344 6255 Email: r.toovey@unimelb.edu.au
Mr John Carey (PhD student) Email: jjcarey@student.unimelb.edu.au

Additional Researchers: Prof Alicia Spittle (University of Melbourne), Prof Christine Imms (University of Melbourne) and Prof Nora Shields (La Trobe University).

Introduction

Thank you for your interest in participating in this research project. The following **three pages** will provide you with further information about the project, so that you can decide if you would like to take part in this research.

Please take the time to read this information carefully. You may ask questions about anything you don't understand or want to know more about.

Your participation is voluntary. If you don't wish to take part, you don't have to. If you begin participating, you can also stop at any time.

What is this research about?

Cycling is a fun physical activity that can be readily adapted for riders of all abilities. To date, research has mainly focused on the best ways to train cycling skills for children and young people with disability who aim to ride a two wheel-bike. Little is known about how to best support cycling opportunities for riders with disability who would like to ride an adapted bike or trike.

For the purpose of this study, by adapted bike or trike, we mean a specialised bicycle or tricycle that caters for the needs of a person with disability. Examples include, an adapted trike with body supports, a recumbent hand-cycle or a standard bike that has been modified with specialist equipment (e.g. larger outriggers/training wheels).

We want to understand more about the people across Australia who support young people and their families to use an adapted bike or trike for cycling. This includes the experiences of occupational therapists (OTs), physiotherapists, teachers, cycling coaches and sports and recreational practitioners, as they often play a leading role in community cycling programs and advising others about cycling.

Supplemental File S4: Pre-information and advertisement material

What will I be asked to do?

Should you agree to participate, you will be invited to complete an online survey which will take **15-20 minutes**. You will be invited to share your experiences working with riders with disability, training adapted cycle skills and if relevant for you, prescribing and/or fitting an adapted bike or trike.

What are the possible benefits?

There are no direct benefits for you in completing this survey.

However, others may potentially benefit from your involvement in this research:

- Your shared knowledge and experience may help to support cycling opportunities for more children and young people with disability.
- This research may help identify factors that are important for training cycling skills and adapting equipment for riders with disability.

What are the possible risks?

There are no anticipated risks associated with taking part in this research project.

We understand that this study carries a time commitment and a need for internet connection which you will not be reimbursed for.

The questions posed in this online survey do not cover stressful or sensitive themes. However, if you are not comfortable responding to a question you can choose to skip that question or take a break and return to it later.

Do I have to take part?

No. Participation is completely voluntary. You can choose to stop taking the survey at any time. If you choose to provide your email address as part of this survey and wish to withdraw your data, you can do so by contacting the responsible researchers.

Will I hear about the results of this project?

If you want to be told about the study findings, you can choose to leave your email address at the end of the survey. At the end of the research project, we may present the results at conferences. We may also publish the results in health journals. We will do this in a way that protects your privacy. This project will form a part of John Carey's PhD research and thesis at the University of Melbourne.

What will happen to information about me?

We will collect data through the **REDCap** survey tool. REDCap is a password protected web-application commonly used in health research. Because providing cycling interventions for riders with disability is a relatively small area of practice, there is a small possibility that your information may be **re-identifiable**. To counteract this risk, where deemed appropriate, we will report data in groups and categories. For example, if you describe or name the company you work for, we would de-identify it by relating it back to the category of industry such as the education sector or disability organisation. Gender will not be collected as part of this study. You may choose to provide your **personal identifying information** (e.g. email address) to be informed of results in the final section of the survey. This identifying information will be kept

private and stored separately in the University of Melbourne's password protected secure server.

As part of our data management plan, only researchers involved in this project may have access to your potentially **re-identifiable information**, which will include:

- Research team involved with this project: who will come from the University of Melbourne and La Trobe University. Professor Nora Shields from La Trobe University will be given access permissions to view de-identified files. All data will be stored on the University of Melbourne server.
- The University of Melbourne Human Research Ethics Committee.

We will keep the project data on a secure server in the University of Melbourne for at least 5 years from the date of final publication. After 5 years, the data will be securely destroyed.

In the interests of advancing science and healthcare, we may share the data collected from this study with other ethically approved research studies and medical journals. In this case, only data from the survey responses will be sent on, and your email address (if provided) will remain in a separate dataset within the University of Melbourne. Extended consent allows us to share data from this study with related projects (e.g. other adapted bike or trike studies) or research in the general areas of disability, assistive technology or cycling. You can choose to provide your **extended** consent by selecting 'yes' in the second 'extended consent' tick box in the survey.

Where can I get further information?

If you would like more information about the project, please contact the researchers; John Carey (Tel: +61492 802824 or Email: jjcarey@student.unimelb.edu.au) or Dr Rachel Toovey (Tel: +613 8344 6255 or Email: r.toovey@unimelb.edu.au).

Who can I contact if I have any concerns about the project?

This research project has been approved by the Human Research Ethics Committee of The University of Melbourne. If you have any concerns or complaints about the conduct of this research project, which you do not wish to discuss with the research team, you should contact the Manager, Human Research Ethics, Research Ethics and Integrity, University of Melbourne, VIC 3010. Tel: +61 3 8344 2073 or Email: HumanEthics-complaints@unimelb.edu.au. All complaints will be treated confidentially. In any correspondence please provide the name of the research team or the name or ethics ID number of the research project.

Supplemental File S4: Pre-information and advertisement material

Email invitation:

Dear [provider/organisation representative]

Have you or others you work with previously supported cycling opportunities for children and young people with disability who use an adapted bike or trike?

Physiotherapy and occupational therapy researchers from the University of Melbourne and La Trobe University are interested in finding out more about how different practitioners support, deliver and recommend adapted bike and trike equipment and training across Australia.

We are inviting occupational therapists, physiotherapists, teachers (special education and mainstream health and physical education), cycling coaches (cycling and triathlon) and sports and recreation practitioners to participate in an online survey lasting 15-20 minutes to document their prior experiences of providing adapted cycling interventions.

To find out more about this survey, check your eligibility and choose to be involved in the study, please follow the following link: REDCAP LINK

If you would like further information about this project please contact Dr Rachel Toovey (email: r.toovey@unimelb.edu.au) or Mr John Carey (email: jjcarey@student.unimelb.edu.au).

We welcome you to share this email with other cycling enthusiasts who work or volunteer in the roles described above and may be able to contribute with their cycling expertise.

John Carey

Graduate Research Student: PhD Candidate (Health Sciences)
Physiotherapist | Research Associate CP-Achieve (MCRI)

School of Health Sciences | The University of Melbourne
Level 6, 161 Barry St, Carlton, Victoria 3010 Australia
Email: jjcarey@student.unimelb.edu.au
Phone: +61 492 802 824

I acknowledge the Traditional Owners of the land on which I work, and pay my respects to the Elders, past, present and emerging.

Supplemental File S4: Pre-information and advertisement material

Social media invitation:

Adaptive Cycling Survey

Researchers from the University of Melbourne and La Trobe University are interested in finding out more about how different practitioners support, deliver and recommend adapted bike and trike assistive technology and training across Australia.

We are inviting allied health practitioners (OTs, physiotherapists, exercise physiologists), teachers, cycling coaches and sports and recreation administrators to take part in an online survey to document their prior experiences of providing adapted cycling interventions.

To find out more about this survey, check your eligibility and choose to be involved in the study, please follow the following link: REDCAP LINK

Contact information: John Carey (jjcarey@student.unimelb.edu.au or 0492802824).

Supplemental File S4: Pre-information and advertisement material

Study recruitment poster:

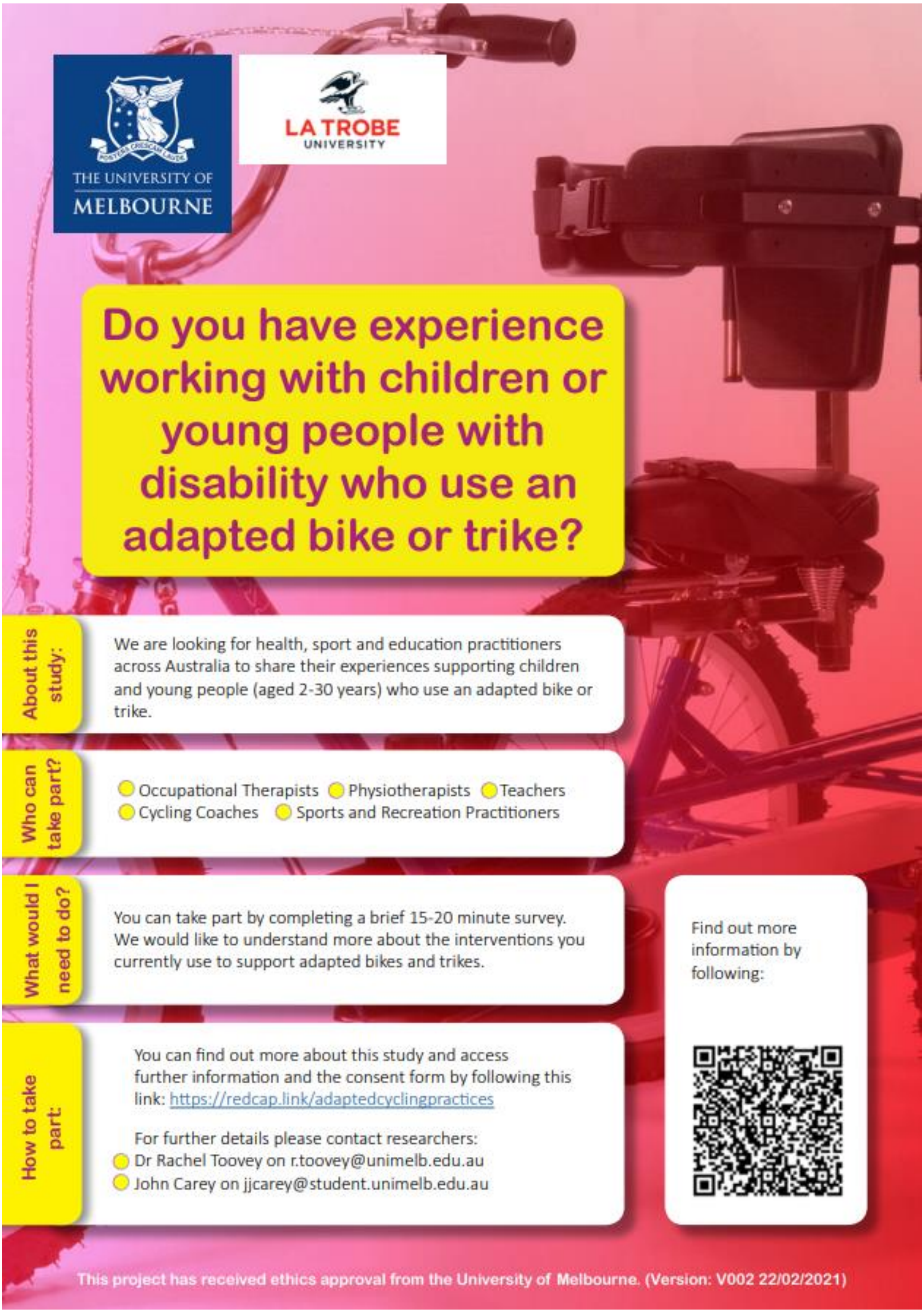

Supplemental File S4: Pre-information and advertisement material

Information sheet on adapted bicycles and tricycles:

Name of adapted bike or trike	*Image of adapted bike or trike*
**Adapted Tricycle**<br><br>**Brief Description:**<br>Three wheeled tricycle. Accommodates different types of supportive features e.g. backrest, wide saddle, attendant push handle. Common commercially available examples: Gomier, BodyCycles Edge Range, TheraPlay Range.<br><br>(**Ref:** Independent Living Centres Australia)	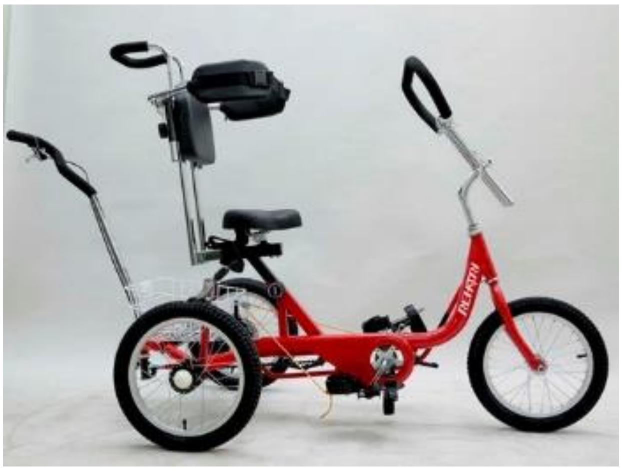<br>*Pictured is a Rehatri Trike with loop handlebar, foot cups, pedal levellers, adjustable backrest, chest or thoracic supports, attendant push-handle.*
**Adapted Bicycle**<br><br>**Brief Description:**<br>Two wheeled bicycle which has specific specialised features to cater for different needs, such as outriggers/training wheels and a low clearance cross bar.<br>Examples; MoMo Therapy bike, Strider Balance Bike.<br><br>(**Ref:** Independent Living Centres Australia)	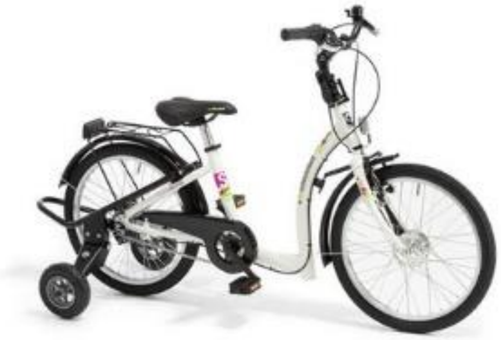<br>*Pictured is the Schuchmann MoMo Therapy Bike with gear set-up and training wheels.*
**Customised Bicycle**<br><br>**Brief Description:**<br>This can include a standard two wheeled bicycle or a specific range carried by a specialist provider (e.g. Freedom Wheels) which has received customised modifications and postural support features based on the rider's needs.<br><br>(**Ref:** Solve-TAD/Freedom Wheels)	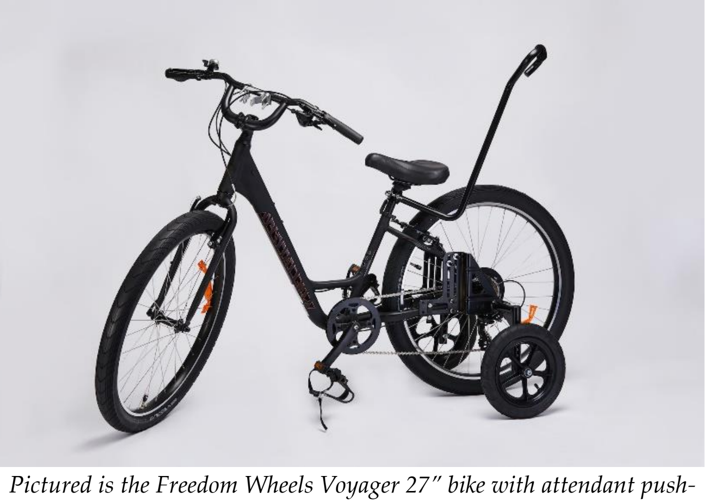<br>*Pictured is the Freedom Wheels Voyager 27" bike with attendant push-handle, wide-set saddle, toe-clips and outriggers.*

Supplemental File S4: Pre-information and advertisement material

**Tandem Bike**<br><br>**Brief Description:**<br>Tandem means one after the other. These bikes can be set up as a standard upright bike or recumbent cycle. **Examples;** CargoCycles Van Raam, Hase Pino AllRound and pictured.<br><br>(**Ref:** Independent Living Centres Australia)	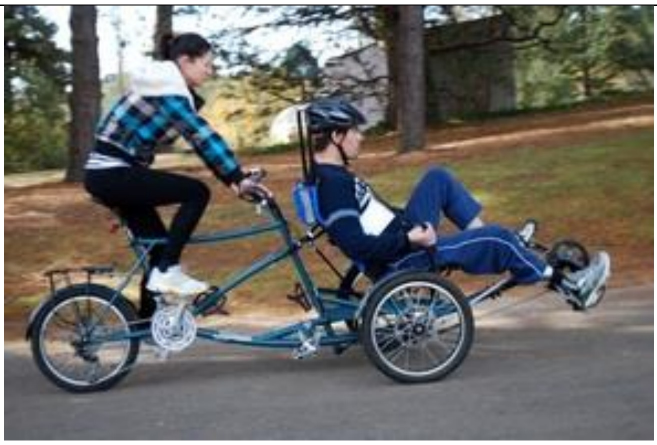<br>*Pictured is a tandem recumbent bike available through Kotzur Mobility Cycles (NSW).*
**Side by Side Bike**<br><br>**Brief Description:**<br>Allows for 2 people to ride alongside each other. **Examples;** BodyCycles Tigermoth.<br><br>(**Ref:** Independent Living Centres Australia)	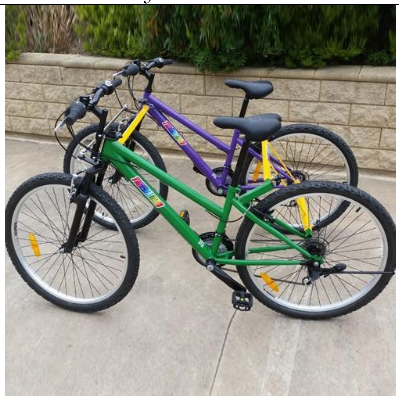<br>*Pictured is the BodyCycles Tigermoth Dual Rider.*
**Tapered Roller Bike**<br><br>**Brief Description:**<br>Adapted bike with rear roller wheel that can gradually be progressed based on rider skill. **Examples:** iCan Ride or Lose the Training Wheel Bikes programs.<br><br>(**Ref:** Temple et al 2016; Klein 2005)	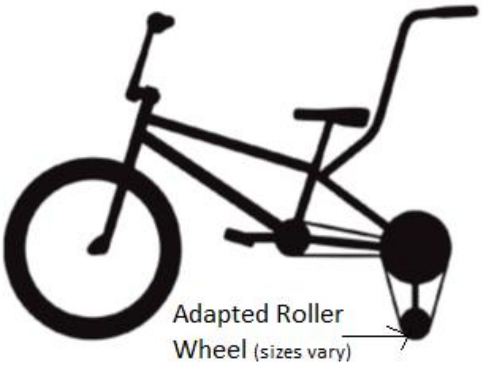<br>*Pictured is a schematic of the adapted roller bike used in iCan Ride Bike Programs.*
**Hand Cycle (Upright Trike)**<br><br>**Brief Description:**<br>Hand propelled system trike with foot rest or leg trough for foot placement. **Examples;** TheraPlay TriLo, TAD Range.<br><br>(**Ref:** Independent Living Centres Australia)	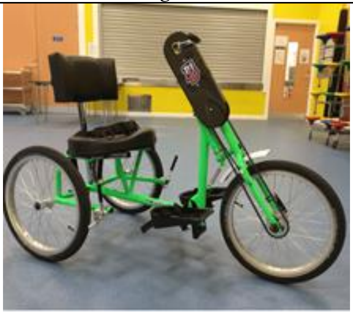<br>*Pictured is a TheraPlay TriLo handcycle with wide-set saddle and backrest support.*

Supplemental File S4: Pre-information and advertisement material

**Recumbent Bike (foot or hand pedal setup)**<br><br>**Brief Description:**<br>Rider pedals from a reclined position and is supported with a backrest and saddle.<br>**Examples;** Quickie Shark Handbike, Rehatri hand cycle, Greenspeed Range.<br><br>(**Ref:** Independent Living Centres Australia)	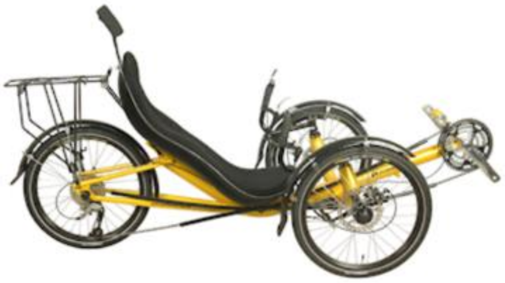<br>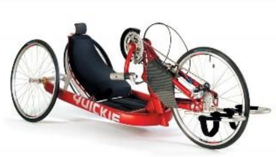<br>*Pictured is a foot pedalled Performer recumbent trike and a Quickie Shark hand-cycle.*
**Wheelchair Cycle**<br><br>**Brief Description:**<br>Attachable add-on which is mounted on to a manual wheelchair and enables hand-cycling.<br>**Examples;** Quickie Attitude Hand Bike Add on.<br><br>(**Ref:** Independent Living Centres Australia)	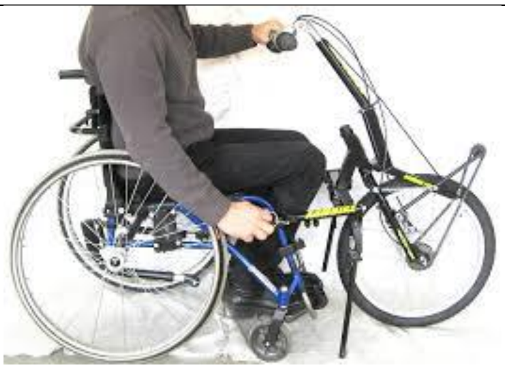<br>*Pictured is a handcycle mount on a manual wheelchair.*
**Trailer or Tag-along Cycles**<br><br>**Brief Description:**<br>The rider is towed on either a trailing cycle attachment or their bike is secured via a mount onto the bike of support person.<br><br>**Examples;** TheraPlay Terrier Hitch, Burley Piccolo trailer, FollowMe attachment.	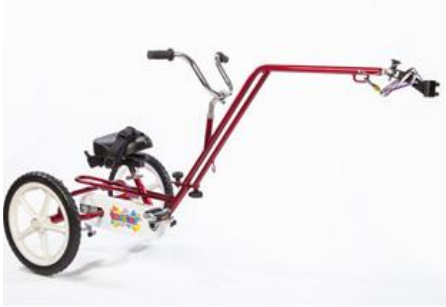<br>*Pictured is the TheraPlay Terrier Hitch.*<br>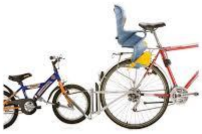<br>*FollowMe attachment to connect bike as tandem attachment.*

Supplemental File S4: Pre-information and advertisement material

<table>
<tr>
<td>E-Bike or Power Add-on

Electric bike or trike power add on feature.

(Ref: Independent Living Centres Australia)</td>
<td>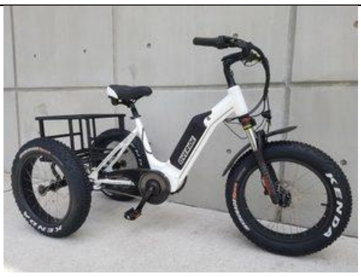
Pictured is the Overide Whipper Electric Trike</td>
</tr>
<tr>
<td>Company Cycle

Brief Description:
Trike or bike is operated by a support person and the young person with disability can enjoy the motion and experience of cycling in their wheelchair or through a tandem seat option.
Examples; Nihola Bike Flex and CargoCycles Van Raam.

(Ref: Independent Living Centres Australia)</td>
<td>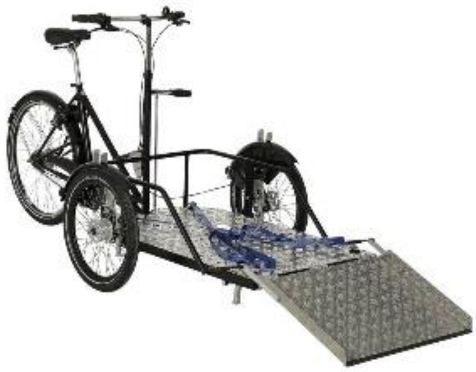
Pictured is a cargo wheelchair bike/trike with ramp attachment in place.</td>
</tr>
<tr>
<td>Prone Recumbent

Brief Description:
Rider lies onto their front and straddle sits on saddle. Pedals are orientated behind the rider.
Examples; Custom made, Freedom Cycles (US).

(Ref: Freedom Concepts)</td>
<td>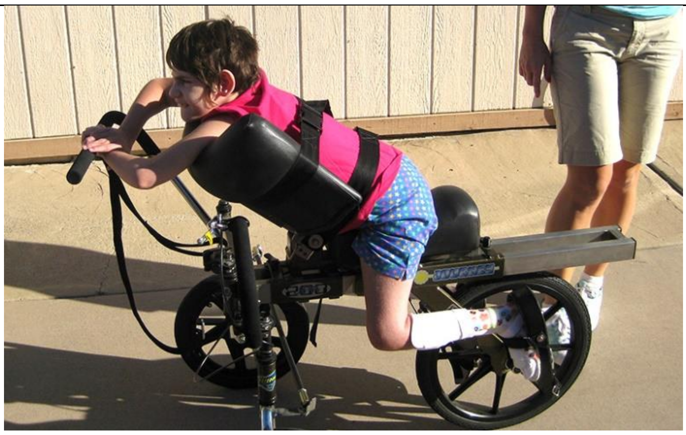
Pictured is a Freedom Concepts Journey Prone Recumbent bike.</td>
</tr>
</table>
