# Supplementary material for: Exploring Adaptive Cycling Interventions for Young People with Disability: An Online Survey of Providers in Australia
Source: J Clin Med. 2023 Aug 25;12(17):5523. doi: 10.3390/jcm12175523 (PMC10488225; doi:10.3390/jcm12175523)
Supplement: Supplementary file 1 [file jcm-12-05523-s001.zip › Supplemental file S5_Resources, Outcome measurements and Participation opportunities_final.pdf]

Supplemental file S5: Resources, outcome measurements and perceived participation opportunities.

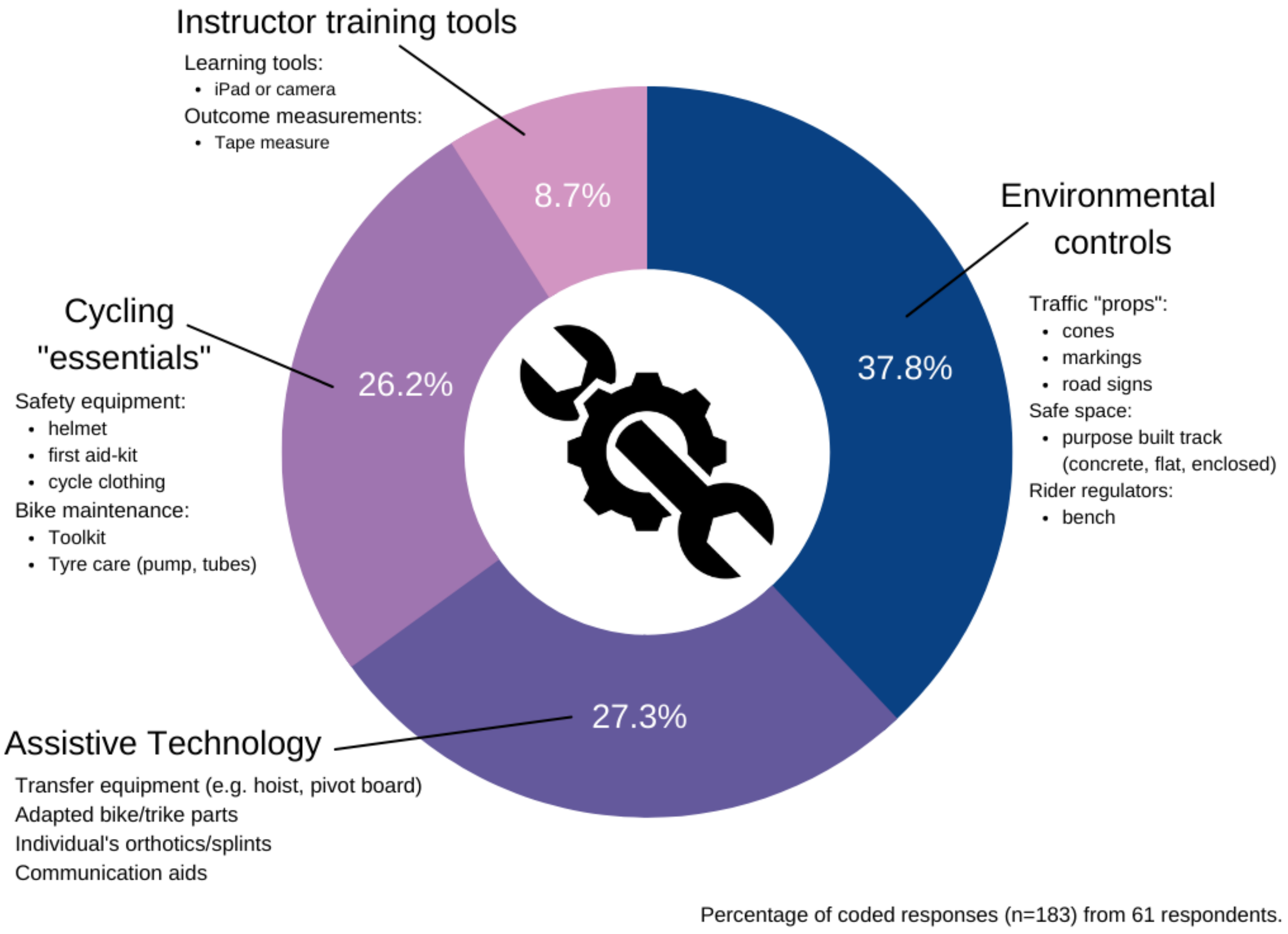

Figure S1: Physical resources used by providers.

Supplemental file S5: Resources, outcome measurements and perceived participation opportunities.

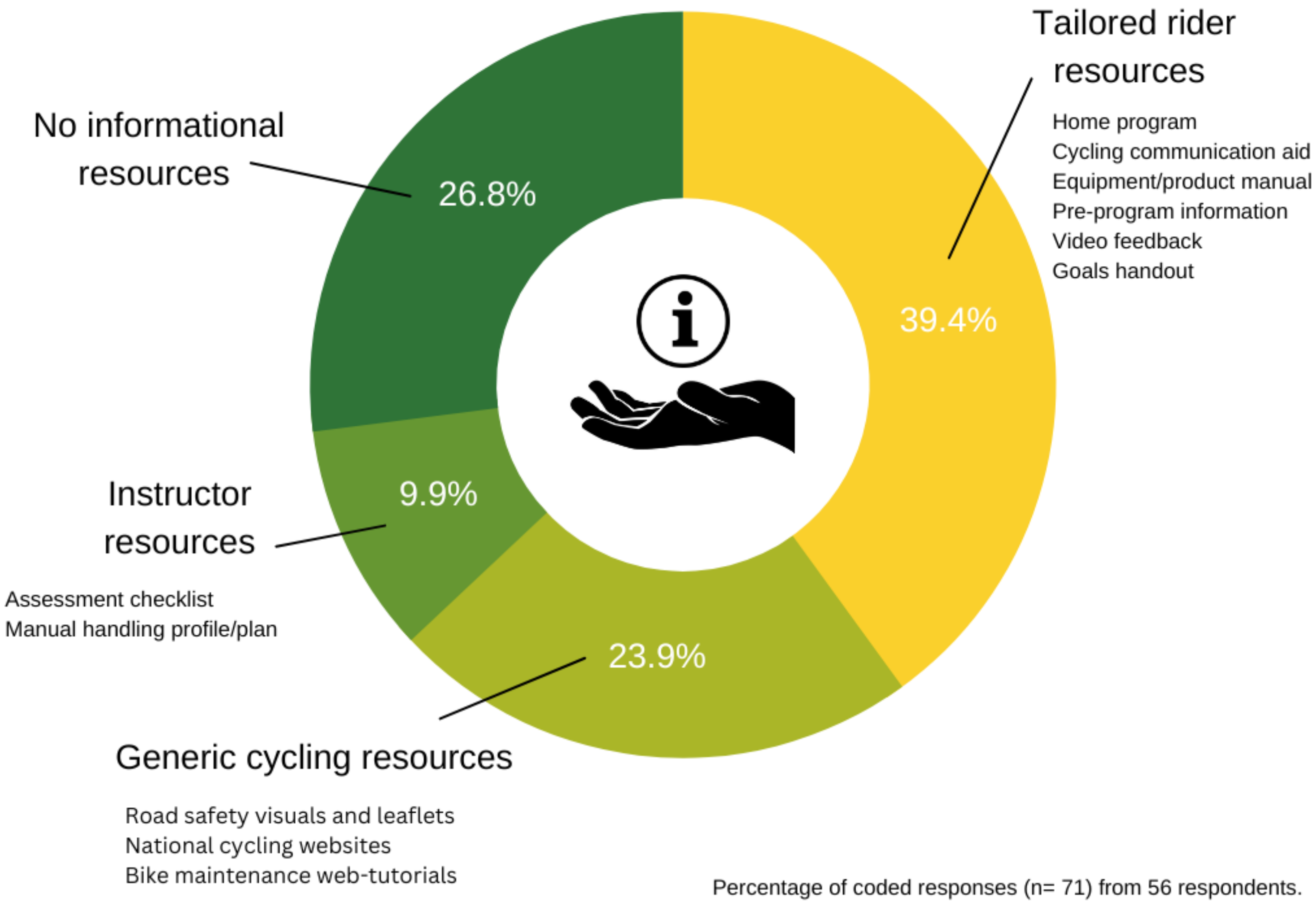

Figure S2: Instructional resources used by providers.

Supplemental file S5: Resources, outcome measurements and perceived participation opportunities.

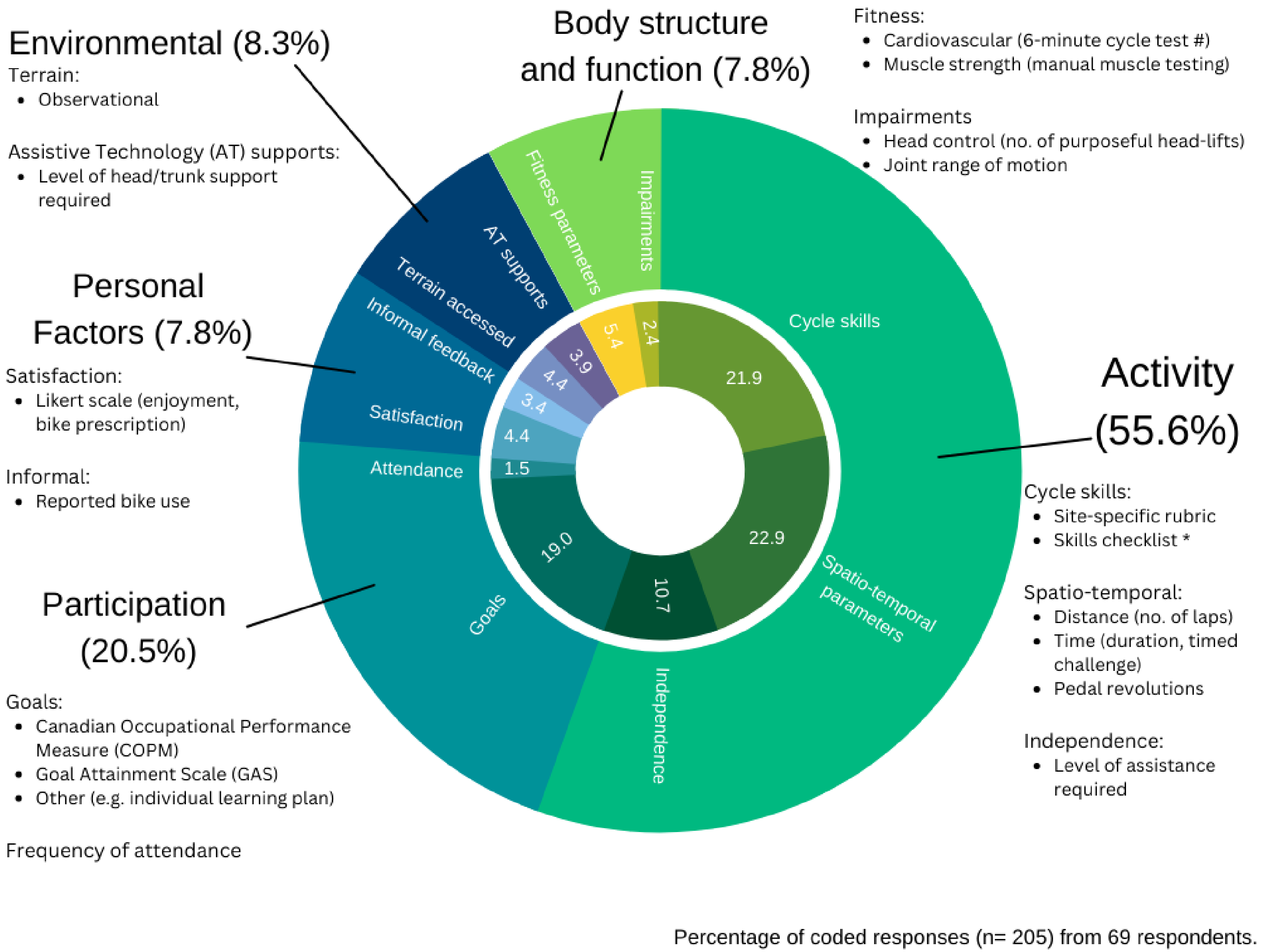

Figure S3: Outcome measurements used by providers. AT supports= assistive technology (i.e. head, upper trunk or lumbar support, attendant handle). *Cycling skills checklist [34]; #Assisted 6-minute cycling test [33]; COPM [31]; GAS [32].

Supplemental file S5: Resources, outcome measurements and perceived participation opportunities.

Table S5. Providers' rating of importance for hypothesized factors to start cycling (i.e. providers' goal)

Factor	Original response	N (%)
Develop strength or fitness [a]	Extremely important	59 (58.4)
	Important	33 (32.7)
	Neutral	8 (7.9)
	Not important	0 (0.0)
	Not important at all	1 (1.0)
Means to play [a]	Extremely important	51 (50.5)
	Important	35 (34.7)
	Neutral	8 (7.9)
	Not important	4 (3.9)
	Not important at all	3 (3.0)
Develop life skills [b]	Extremely important	36 (36.0)
	Important	42 (42.0)
	Neutral	21 (21.0)
	Not important	1 (1.0)
	Not important at all	0 (0.0)
Access sport [a]	Extremely important	43 (42.6)
	Important	31 (30.7)
	Neutral	19 (18.8)
	Not important	3 (3.0)
	Not important at all	5 (4.9)
Community mobility device [a]	Extremely important	33 (32.7)
	Important	30 (29.7)
	Neutral	21 (20.8)
	Not important	13 (12.9)
	Not important at all	4 (3.9)
Tool for rehabilitation [c]	Extremely important	23 (23.2)
	Important	38 (38.4)
	Neutral	18 (18.2)
	Not important	7 (7.1)
	Not important at all	13 (13.1)
Tool for learning to ride a two-wheel bicycle [c]	Extremely important	10 (10.1)
	Important	32 (32.3)
	Neutral	29 (29.3)
	Not important	15 (15.2)
	Not important at all	13 (13.1)

Footer: [a] n=101; [b] n=100; [c] n=99

Supplemental file S5: Resources, outcome measurements and perceived participation opportunities.

Table S6. Providers' rating of importance for hypothesized factors that lead a rider to stop adaptive cycling.

Factor	Original response	N (%)
Change in rider's function [(a)]	Extremely important	48 (51.1)
	Important	36 (38.3)
	Neutral	8 (8.5)
	Not important	2 (2.1)
	Not important at all	0 (0.0)
Rider motivation [(b)]	Extremely important	51 (53.7)
	Important	33 (34.7)
	Neutral	10 (10.5)
	Not important	1 (1.1)
	Not important at all	0 (0.0)
Informal opportunities [(b)]	Extremely important	41 (43.2)
	Important	40 (42.1)
	Neutral	10 (10.5)
	Not important	2 (2.1)
	Not important at all	2 (2.1)
Transport of adapted cycle [(b)]	Extremely important	51 (53.7)
	Important	26 (27.4)
	Neutral	11 (11.6)
	Not important	3 (3.1)
	Not important at all	4 (4.2)
Access to skilled provider [(a)]	Extremely important	22 (23.4)
	Important	47 (50.0)
	Neutral	17 (18.0)
	Not important	4 (4.3)
	Not important at all	4 (4.3)
Cost [(b)]	Extremely important	36 (37.9)
	Important	23 (24.2)
	Neutral	23 (24.2)
	Not important	9 (9.5)
	Not important at all	4 (4.2)
Transition period [(a)]	Extremely important	23 (24.4)
	Important	34 (36.2)
	Neutral	32 (34.0)
	Not important	1 (1.1)
	Not important at all	4 (4.3)
Formal opportunities [(b)]	Extremely important	24 (25.3)
	Important	30 (31.6)
	Neutral	22 (23.1)
	Not important	11 (11.6)

Supplemental file S5: Resources, outcome measurements and perceived participation opportunities.

	Not important at all	8 (8.4)
Repairs or maintenance (b)	Extremely important	23 (24.2)
	Important	29 (30.5)
	Neutral	23 (24.2)
	Not important	11 (11.6)
	Not important at all	9 (9.5)

Footer: (a) n=94; (b) n=95

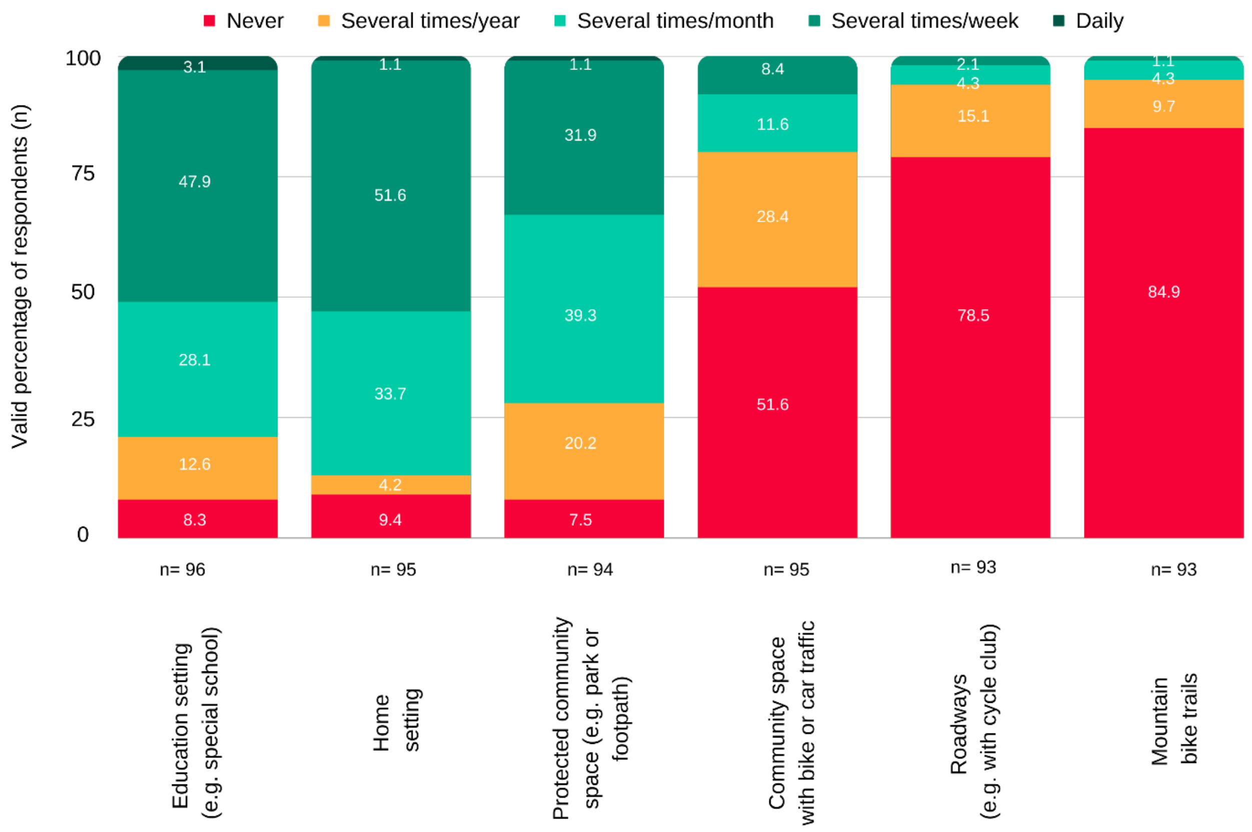

Figure S4: Frequency of perceived participation in different settings.

Supplemental file S5: Resources, outcome measurements and perceived participation opportunities.

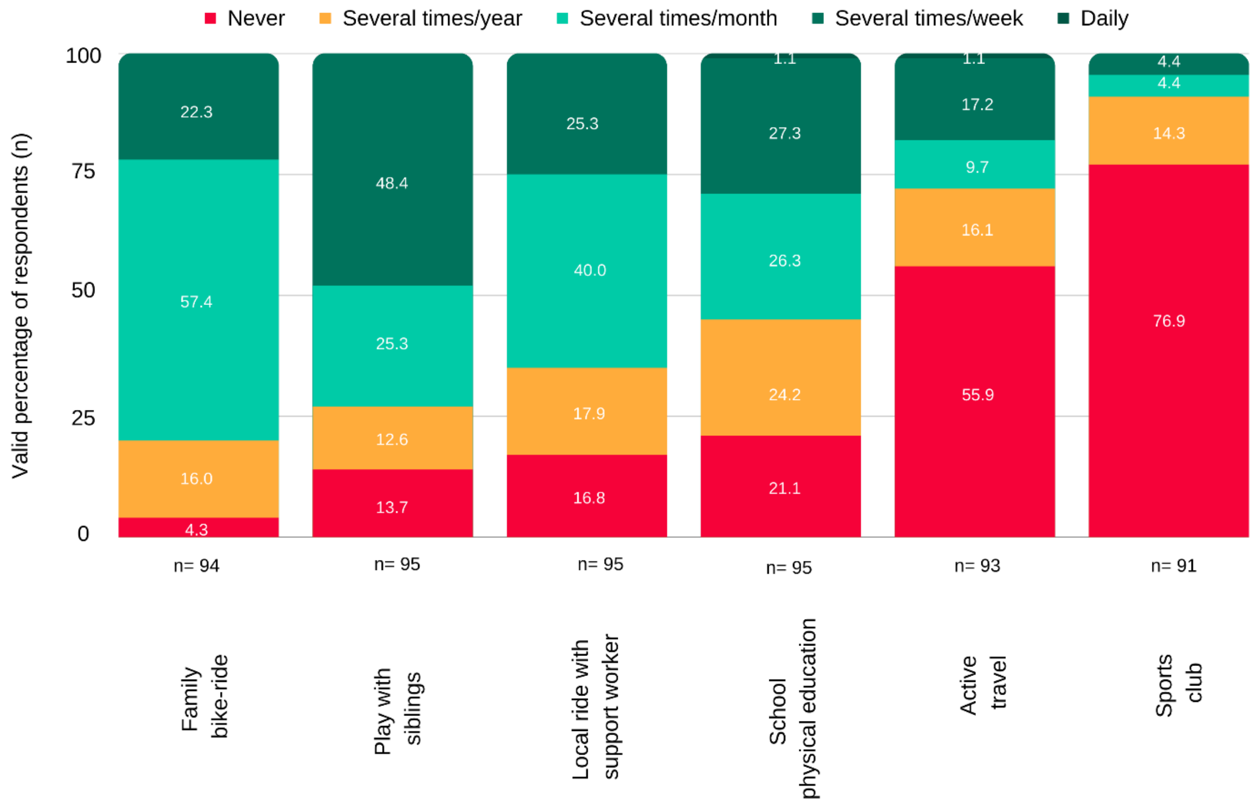

Figure S5: Frequency of perceived participation in different activities.
