# Supplementary material for: Exploring Adaptive Cycling Interventions for Young People with Disability: An Online Survey of Providers in Australia
Source: J Clin Med. 2023 Aug 25;12(17):5523. doi: 10.3390/jcm12175523 (PMC10488225; doi:10.3390/jcm12175523)
Supplement: Supplementary file 1 [file jcm-12-05523-s001.zip › Supplemental file S6_ Provider perceived barriers and facilitators_final.pdf]

Supplemental File S6: Barriers and Facilitators Table

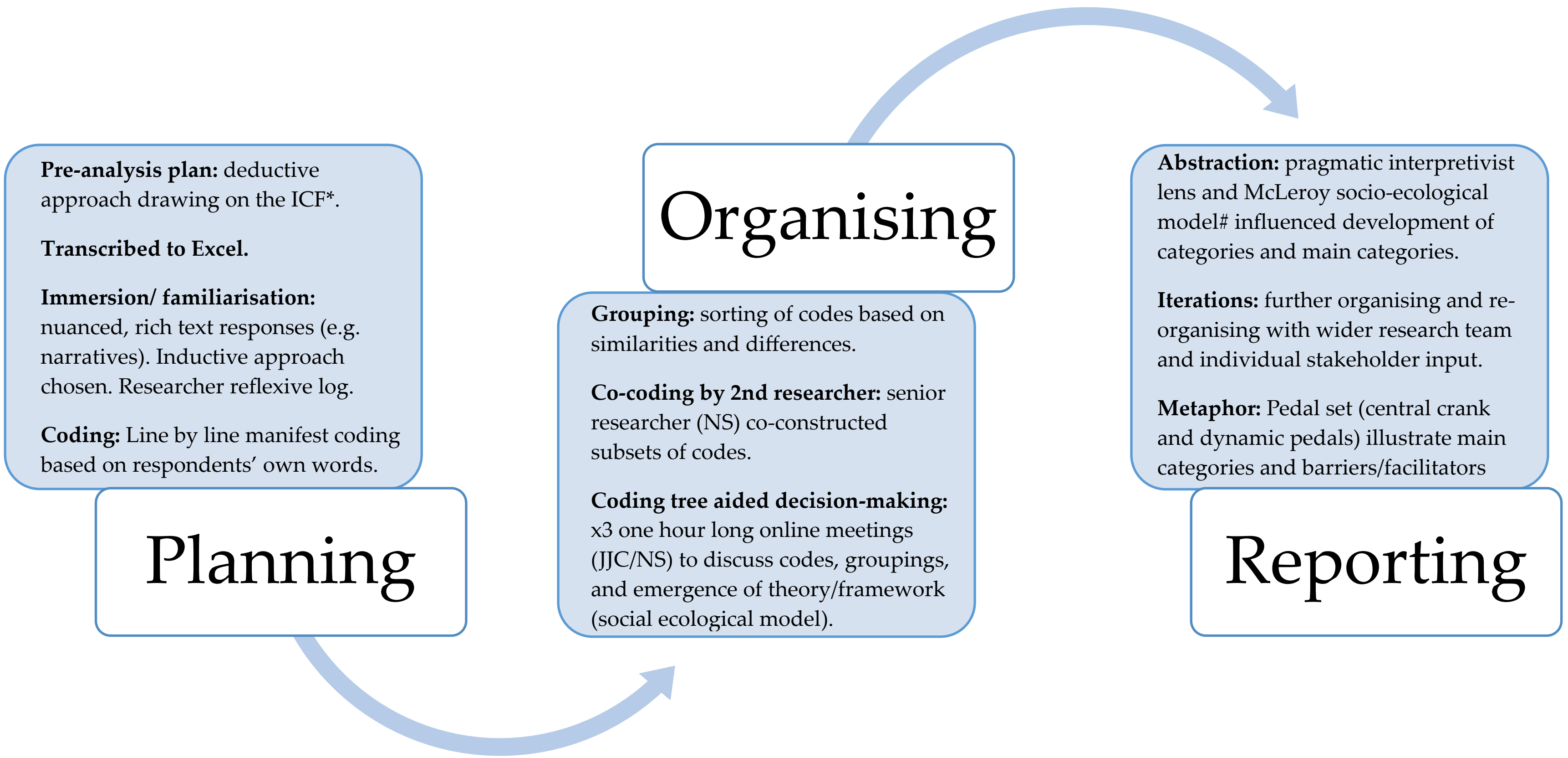

Figure S6: Qualitative methods (inductive content analysis) and audit trail overview for open-ended reflective responses.

*ICF: International Classification of Functioning, Disability and Health. World Health Organization. (2001). International classification of functioning, disability and health : ICF. World Health Organization. https://apps.who.int/iris/handle/10665/42407

#McLeroy's socioecological model of health [36]

Supplemental File S6: Barriers and Facilitators Table

Table S7: Coding tree from the qualitative analysis of barriers and facilitators.

Main Categories	Categories	Sub-categories	Citations
**Rider-related factors**	"Ready to train"	**Facilitators**<br>+ Motivated riders<br>+ Enjoys cycling<br>+ "Coachable attributes": demonstrated ability (physical and cognitive)	"desire to ride is biggest factor" P52<br>"Level of satisfaction when they can actually ride" P24<br>"Motivation- young adult who wanted to ride to work" P125<br>"many of our Autistic students … like that social interaction of participating in the same activity as other students and being alongside them" P58<br>"Rider related abilities" P67<br>"Physical ability to ride (not too difficult or painful)." P122<br>"achieve the pedal motion" P24
		**Barriers**<br>- Decline in priorities and interests<br>- Negative experience of cycling (a fall/ crash, rider "feeling different")<br>- Unable to "keep up with peers"	"Grow out of it- develop other interests." P102<br>"A crash. …participants may get a fright while learning if there is even a minor crash; this can be a barrier to resuming riding." P29<br>"Not being able to keep up as siblings or peers progress on to more advanced cycling." P51<br>"Bike looks too 'disability-looking' compared to peers, child gets embarrassed and stops wanting to participate". P18
	Challenging support needs	**Facilitators**<br>+ Accommodated with supports (assistive technology + people-based)	"Everyone is able to [cycle], it just depends on the level of support needed and the type of bike needed"- P37
		**Barriers**<br>- Physical complexity too challenging to accommodate	"head control – [sustaining a] safe position… and tone - extreme dystonic movements with an inability to be strapped in… limiting safety or ability to participate" P12

		- Deterioration in function (with age, pain, weight-gain or surgery)<br>- Deterioration in health (epilepsy, progressive condition)<br>- Displays behaviours of concern around foot, bike or car traffic	"[changes in] … cerebral palsy with increased spasticity, increasing contractures and general declining ability to move." P95<br>"usually due to discomfort while riding, which at times cannot always be conveyed by the user" P107<br>"Behaviours of concern exhibited by child that puts them and helpers at risk" P57<br>"For older children [functioning at] Gross Motor Function Classification System [Level] IV or V transfers … become more of an issue." P30<br>"Physical ability change and no longer able to complete safe transfer" P122<br>"Deterioration of medical condition impacting on physical ability (regardless of willingness and enthusiasm)." P9<br>"inability to follow safety instructions so need close adult supervision at all times on the bike/trike" P4
**Peloton of supports**	"Someone to ride with" (P41)	**Facilitators**<br>+ Family engaged in community practice (involved, invested, priority, leads)<br>+ Access to companion rider (support worker or peer) for community<br>+ "Making it a family adventure"	"making it a family adventure" P2<br>"Supportive family members ...who lead and provide ample opportunities for the rider to use their trike" P120<br>"Low family enthusiasm" P11
		**Barriers**<br>- Family "disengagement" (priorities change, motivation, time)<br>- Link lost to formal supports	"not enough additional practice at home" P52<br>"not having anyone to take them riding" P74<br>"difficulties finding experienced coaching" P79
	Network's capacity to support	**Facilitators**<br>+ Access to specialist allied health assessment (advocate, initial assessment, goals)	"Skilled practitioner" P62<br>"trained staff to know how to assess a rider and implement the right type of assistive technology at the right time" P71

	+ Access to cycling suppliers and mechanics<br>+ Allied health knowledge on adapted cycles (funding, matching bike frame to goal, function and terrain)<br>+ Formal training on bike use and practice ("implementation")<br>+ Cohesion between supports and sectors (goal-sharing, equipment trial between sectors, joint cycle plan)	"Professionals need experience of what adaptive bikes are around and the potential of people to use them. They need to clearly understand the benefits and pitfalls of each bike and how it relates to the individuals goals, or risk prescribing something inappropriate." P79<br>"…having someone advocating for them and organising an assessment" P19<br>"… having the commitment or time provided by family members... Families have to be very interested and keen to find out what sort of bike suits the student." P61<br>"Staff and parent training on how to support use of bike is integral." P112<br>"… therapy staff that LINK with the school too and work on shared goals in cycling or advise on cycling equipment" P89<br>"Shared support…" P101<br>"Knowledgeable and experienced suppliers or technicians at local suppliers". P60
	**Barriers**<br>- Transfers too unsafe to support (manual handling demand, hoist)<br>- Providers lacking knowledge in cycling options (AT, accessible routes, community initiatives)<br>- Poor adapted bike choice (generic frame, unsuited to purpose)<br>- Poor monitoring of rider growth (musculoskeletal and developmental)	"Effort required as they get bigger, but not necessarily stronger and if it becomes laboursome to get on/off, e.g. Reliant on a hoist to transfer" P36<br>"Knowing what is available… lack of awareness of [adapted cycle] options… where to ride… who to ride with (friends, groups etc..)" P79<br>"Adaptation needed to make the bike 'work' and nobody identifies this, so bike sits in garage collecting dust." P81<br>"many kids get given a fairly universal adaptive trike when younger". P21<br>"Most often, the adapted bike that was prescribed for them was not suited to their cycling goals" P41<br>"...poor fit. Bad trike choice for intended use". P79<br>"Outgrown and not followed up or reported" P69<br>"Bike not being reviewed and changed to meet their needs or skill progression..." P97

Supplemental File S6: Barriers and Facilitators Table

	"Peoples' perceptions and expectations"	**Facilitators**<br>+ Identity as a "cycling family"	"Proactive and active family. Cycling important to family" P59
		**Barriers**<br>- Cycling only achievable in traffic-free areas<br>- Cycling goals are "too much trouble"	"Families perceive child isn't interested or ready" P54<br>"It's not an option parents consider possible" P49<br>"Parent perspective - not important, too much trouble" P7<br>"Don't see many adaptive bikes out in the community" P39<br>"cycling generally in the community is perceived to be unsafe" P81
**"Equipment and opportunities"**	Access to a "suitable" bike	**Facilitators**<br>+ Owns or loans an adapted bike<br>+ Ability to try different bikes<br>+ Well maintained bikes<br>+ Bike features match needs, goals and proposed use<br>+ Bike features are easy to use and move for others (attendant steering, weight of bike).	"a suitable bike to suit the abilities of the rider" P117<br>"well-prescribed bike with correct adaptations that meet the child's unique needs and circumstances" P81<br>"Multiple bikes to trial" P110<br>"Schools [have a] large range [of adapted bikes] available for sustained trial, easy to change to another option or modify if needed, lots of parts to adapt tricycles further when needed" P35<br>"Ease of use = increased frequency of use!" P 112
		**Barriers**<br>- Loses access to adapted bike (loan>own, influenced by growth, transition, maintenance)<br>- Trike hits the "tipping point" for staff and riders (i.e. unsafe to push, rider tips on turning)	"the bike breaks and it doesn't get fixed …it gets put in the too hard basket." P86<br>"Without access to adaptive bike specific to the individuals needs it very difficult for children with disabilities to access and have the opportunity to develop skills in cycling" P18<br>"…the trike becomes tippy and harder to control and the support worker finds it too hard. Instead of looking for a more appropriate cycle they abandon this activity." P21<br>"Size of trike and inconvenience of it being transported in car for use." P80<br>"When the bike no longer suits the persons anthropometrics it often results in abandonment" P107

Supplemental File S6: Barriers and Facilitators Table

	Opportunities to experience and practice	**Facilitators**<br>+ Regular opportunities to ride locally (informal)<br>+ Access to bike-riding in school<br>+ Access to formal skills programs<br>+ Positive learning experience	"Opportunities for regular, fun rides with associated social interactions" P7<br>"regular opportunities to experience and practice bike riding" P100<br>"Opportunities at school to learn to cycle" P96<br>"Time [spent] on the bike with support" P34
		**Barriers**<br>- Community clubs, programs and events are lacking<br>- Existing programs prioritise bicyclists	"No inclusive groups or events P69<br>"Inclusion criteria for school holiday cycling programs require the child to have the goal to ride a two-wheeled bike" P41
**Cycling environment**	"Nowhere to go" safely	**Facilitators**<br>+ Immediate local access to safe spaces without traffic (car>bike>foot traffic)<br>+ Flat, concreted, protected areas with wide spaces to practice	"safe place to ride the bike away from traffic" P47<br>"Local opportunities = motivation to persist and greater carry-over into 'real life'" P36<br>"Easy access to the community- local courts or recreational reserves and bike tracks, driveways" P50<br>"Appropriate and engaging environments and contexts to use the bike or trike in". P26<br>"Flat community areas for them to learn to pedal and manoeuvre" P38
		**Barriers**<br>- Unsafe local route featuring car-traffic<br>- Lack access to providers and suppliers for regional and remote communities<br>- Hilly, uneven, terrain	"limited to around home and surrounds (may not be safe around roads etc)" P4<br>"location of living - rural vs metro" P12<br>"Living regionally, there are limited adaptive tricycle companies around… Families need to travel over 2.5 hours to assess tricycles in [the nearest major city]…." P120<br>"Geography - hilly, dirt roads". P7

Supplemental File S6: Barriers and Facilitators Table

	"Built environment isn't suitable"	**Facilitators**<br>+ Purpose-built safe cycling amenities (e.g. footpaths, specialist schools)<br>+ Facilities to store an adapted cycle<br>+ Access to transport (trailer, utility vehicle) to reach traffic free areas	"School resources (e.g. we have an excellent track at our school)" P91<br>"Well developed footpaths (away from traffic) in the local area" P55<br>"Ability to transport a modified bike" P97<br>"requires a trailer or a ute" P29
		**Barriers**<br>- Physical barriers within cycling route<br>- Cycling infrastructure absent in some regional and remote areas<br>- No storage space at home	"The community-built environment is not suitable for adapted bikes (e.g. tight chicanes, narrow or no ramps, aggressive camber, steep hills, physical barriers etc. or pathways not simply built "to code")" P81<br>"some suburbs …do not have foot paths." p111<br>"nowhere to store bike at home" P35
**Policies to pedal**	"Inconsistencies" with funding	**Facilitators**<br>+ Attain funding approval to implement cycling plans from prescription to participation.	"approval of funding for involvement in cycling specific programs/prescription of equipment, sufficient support worker funding to assist with community, social and recreational participation" P28
		**Barriers**<br>- Rejection, delays and uncertainty from inconsistent funding decisions<br>- Process burden too high (time and administrative load)	"…rejected…on the criteria 'it does not fit diagnosis'" P38<br>"funding… is inconsistent even when a need is demonstrated" P60<br>"Having to wait for assessment, application and funding process where able bodied children can commence riding once the thought is had" P51
	Cycling valued & prioritised	**Facilitators**<br>+ Decision-makers understand the impact of cycling on goals.<br>+ School leadership prioritises cycling within special education	"Understanding from funding bodies that cycling] can be used for a number of functional goals" P33<br>"…allocated into [the school] timetable" P48

Supplemental File S6: Barriers and Facilitators Table

		**Barriers**<br>- Cycling budget cut due to costs	"other priority AT devices rather than leisure device…" P118<br>"…less staff to supervise or changed programs and altered environment. Equipment breaks and is not replaced or repaired due to cost or time." P100<br>"… 1 to 1 adult to student ratio is best but not achievable due to budget constraints" P58

List of codes that did not meet data saturation (i.e. <5 responses across the 4 data sets analysed):

Rider-related factors:

- Rider's mental wellbeing
- Fear of seizures

Peloton of supports:

- Strong family advocacy
- Parents project fears
- Cycling culturally unimportant
- Inexperienced family cyclists
- Network loses interest

Cycling equipment and opportunities:

- Competitive races lacking
- Power-assist tackles terrain

Cycling environment:

- Unhelpful community attitudes
- Inclement weather

Policies to pedal:

- Cycling laws differ
- COVID-19 crisis
